# Sympathetic signaling activation alleviated acute respiratory distress syndrome via the HDC/SLC7A11 axis in lipopolysaccharide-induced macrophages

**DOI:** 10.1038/s41420-026-03173-0

**Published:** 2026-06-15

**Authors:** Xing Lv, Chenhao Jiang, Xu Zhang, Zhengqi Wu, Xuxia Wei, Yang Zhao, Jianhao Zhang, Xuegang Zhao, Lu Han, Yufeng He, Jianrong Liu, Yujun Zhang, Yuling An, Xiaomeng Yi, Yingcai Zhang, Xin Sui, Huimin Yi

**Affiliations:** 1 Department of Surgical Intensive Care Unit, The Third Affiliated Hospital, Sun Yat-sen University, Guangzhou, China; 2 Guangdong Key Laboratory of Liver Disease Research, The Third Affiliated Hospital, Sun Yat-sen University, Guangzhou, China; 3 Department of Hepatic Surgery and Liver Transplantation Centre, The Third Affiliated Hospital, Sun Yat-sen University, Guangzhou, China; 4 Department of Gynecology, The Third Affiliated Hospital, Sun Yat-sen University, Guangzhou, China; 5 Department of Vascular Surgery, The Third Affiliated Hospital, Sun Yat-sen University, Guangzhou, China; 6 Hepatobiliary Pancreatic Center, People’s Hospital of Xinjiang Uygur Autonomous Region, Urumqi, China

**Keywords:** Cell death and immune response, Cell death, Immune cell death

## Abstract

Acute respiratory distress syndrome (ARDS) represents a severe pulmonary condition characterized by excessive inflammation, wherein alveolar macrophages (AMs), pivotal components of the innate immune system, play a critical role in the pathogenesis of the disease. Despite its high morbidity and mortality, effective targeted therapies for ARDS remain unavailable. Norepinephrine (NE) is an endogenous neurotransmitter with immunomodulatory and anti-inflammatory properties, and has been reported to mitigate ARDS symptoms in sepsis models. While sympathetic signaling exerts protective effects, the underlying immunomodulatory mechanisms-especially those involving macrophages-remain poorly defined. Our in vitro experiments demonstrated that NE confers protection against LPS-induced injury in AMs by limiting lipid peroxidation, sustaining mitochondrial integrity, and upregulating antioxidant regulators SLC7A11 and GPX4, leading to improved cell viability. Mechanistically, the anti-ferroptotic effect of NE on LPS-treated AMs was significantly impaired by β2-adrenergic receptor (β2-AR) blockade or knockdown of histidine decarboxylase (HDC). Our in vivo experiments further demonstrated that salbutamol, a selective β2-AR agonist, upregulated SLC7A11 and GPX4 expression in septic mice and concurrently increased HDC expression in AMs. Furthermore, salbutamol alleviated lipid peroxidation, mitigated macrophage and lung tissue injury. These findings identify HDC/SLC7A11 as a potential axis involved in the neuroimmune regulation of ferroptosis in AMs, offering a potential therapeutic target for ARDS.

## Introduction

Acute respiratory distress syndrome (ARDS) is a critical clinical condition characterized by bilateral pulmonary infiltrates and severe hypoxemia resulting from noncardiogenic pulmonary edema [1, 2]. Despite substantial advances in supportive care and mechanical ventilation strategies, ARDS is still associated with high morbidity and mortality rates [3–5]. Among various immune populations, alveolar macrophages (AMs), as key innate immune sentinels of the pulmonary microenvironment, have been recognized to orchestrate the initiation and propagation of inflammatory responses during ARDS [6, 7]. Our previous studies have shown that targeted modulation of the macrophage cholinergic anti-inflammatory pathway (CAP) can effectively alleviate lung injury in ARDS, indicating a mechanistic link between cholinergic neural signaling and macrophage-mediated innate immune regulation [8]. Our previous data further suggested that sympathetic signaling may also contribute to these neuroimmune effects. However, it remains unknown whether AMs are influenced by sympathetic neuroregulation in a manner that mitigates lung injury.

Norepinephrine (NE), a major sympathetic neurotransmitter of the sympathetic nervous system (SNS), exerts diverse biological effects through activation of α- and β-adrenergic receptors. Although many previous reports have clarified that sympathetic signaling certainly affects the immune systems, the direction of its immunologic regulation remains uncertain and varies according to the disease situation and responsible cell types. For instance, physiological or transient NE activation can be tissue-protective and dampen inflammation, whereas chronic stress or sustained systemic catecholamine exposure is associated with immune dysregulation and enhanced inflammation [9]. Studies have shown that NE modulates cellular metabolism, suppresses inflammatory responses, and promotes cell survival pathways via β2-adrenergic receptor (β2-AR) signaling [10, 11]. Furthermore, the β2-AR is the major subtype expressed on immune cells, particularly macrophages [12]. Taken together, these findings raise the possibility that NE exerts anti-inflammatory effects on AMs through β2-AR signaling during ARDS [13]. Nonetheless, further investigation is required to clarify the specific molecular pathways involved.

The downstream molecular mechanisms by which AMs contribute to sustained tissue damage and disruption of the alveolar–capillary barrier remain incompletely understood. In this context, growing evidence indicates that ferroptosis-a regulated cell death modality driven by iron overload and excessive lipid peroxidation-substantially influences macrophage behavior in inflammatory conditions [14, 15]. However, whether sympathetic signaling modulates macrophage ferroptosis during ARDS remains largely unknown. Ferroptosis is mechanistically distinct from alternative types of programmed cell death and is chiefly defined by iron-catalyzed lipid oxidation and morphological alterations of mitochondria, such as shrinkage and cristae loss, while maintaining nuclear integrity [15]. Increasing evidence highlights ferroptosis as a pivotal contributor to the pathogenesis of various inflammatory and degenerative diseases [16–18], including intestinal inflammation [19] and endothelial injury [20]. Moreover, recent studies suggest that ferroptosis may underlie key pathological mechanisms in chronic respiratory conditions such as COPD, asthma, and other lower airway inflammatory diseases [21]. Recent studies have shown that AMs not only orchestrate inflammatory responses but also undergo ferroptosis themselves, thereby exacerbating lung injury [22]. Moreover, inhibition of macrophage ferroptosis has been reported to ameliorate sepsis-induced acute lung injury and attenuate pulmonary inflammation [23]. These findings underscore the central role of macrophages at the intersection of inflammation and ferroptosis and raise the possibility that targeting macrophage ferroptosis could modify the course of ARDS.

Recent studies have suggested that norepinephrine signaling may influence ferroptosis [24, 25]. For example, norepinephrine depletion in a Parkinson’s disease model, induced by paraquat and maneb administration, has been associated with hippocampal iron accumulation and activation of ferroptotic signaling pathways [26]. By contrast, norepinephrine itself has been reported to exacerbate ferroptosis through disruption of iron homeostasis and suppression of GPX4 [25]. Furthermore, in a model of chronic psychological stress, sustained elevation of norepinephrine has been reported to induce Sertoli cell ferroptosis, characterized by iron overload, lipid peroxidation, and reduced GPX4 expression [27]. These seemingly paradoxical findings indicate a complex and context-dependent role of norepinephrine in ferroptosis regulation. Herein, we selectively activated sympathetic β2-AR signaling in AMs and, in an LPS-induced ARDS model, found that this activation was associated with suppression of ferroptosis and attenuation of pulmonary inflammation. These findings also raise the possibility that this pathway could be explored as a potential therapeutic target.

## Results

### Norepinephrine suppressed inflammation and oxidative stress in AMs

**Fig. 1:**
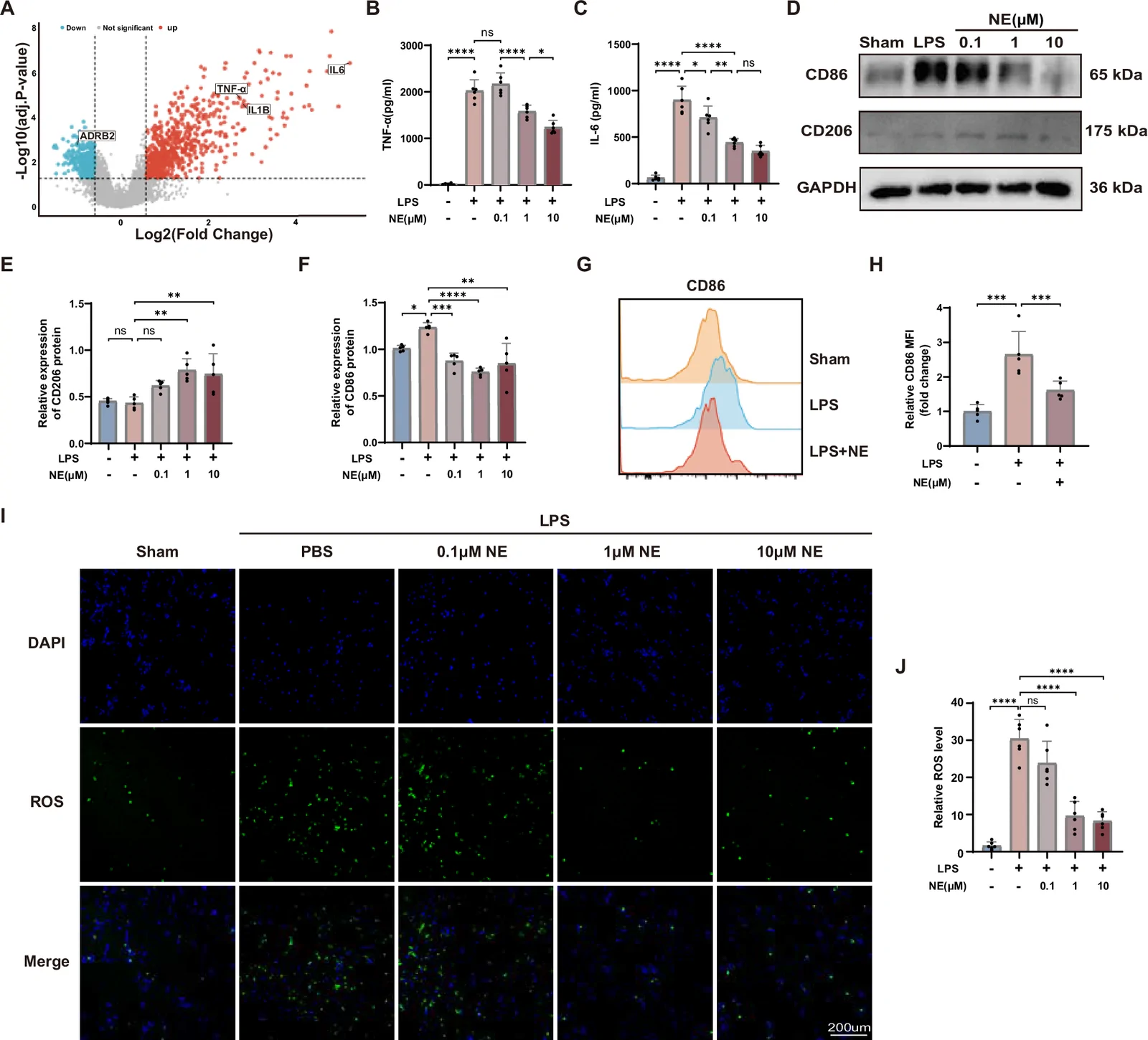
Norepinephrine suppressed inflammation and oxidative stress in AMs. **A** Volcano plot of DEGs in GEO dataset (blue color indicates down-regulated DEGs and red color indicates up-regulated DEGs). **B** TNF-α levels in AMs (*n* = 6). **C** IL-6 levels in AMs (*n* = 6). **D** Representative Western blotting images of CD86, CD206 and GAPDH in AMs. **E** The protein expression of CD206 relative to GAPDH (*n* = 5). **F** The protein expression of CD86 relative to GAPDH (*n* = 5). **G** Representative flow-cytometry histogram showing CD86 expression MFI gated on F4/80^+^ AMs under the indicated treatments. **H** Quantification of CD86 MFI within F4/80^+^ AMs (*n* = 5). **I** Representative images of fluorescence probe for ROS in AMs. Scale bar: 200 μm. **J** Quantification of intracellular ROS fluorescence intensity in AMs (*n* = 6). Data are shown as mean ± SD. Two-way ANOVA with Tukey’s post hoc test was used for analysis. ns not significant; *p < 0.05; **p < 0.01; ***p < 0.001; ****p < 0.0001.

Our previous transcriptomic analysis [8] of murine lung tissues revealed that neural regulatory pathways, particularly sympathetic and parasympathetic signaling, may participate in modulating inflammatory responses. Consistently, analysis of a GEO dataset from human AMs revealed differential expression of sympathetic signaling–related genes after LPS stimulation (Fig. 1A), indicating a potential role of sympathetic pathways in regulating macrophage inflammatory responses. To functionally validate this hypothesis, we isolated primary AMs from BALF. Purity of isolated AMs was confirmed by Cytospin staining, F4/80 immunofluorescence, and flow-cytometric analysis of Siglec-F⁺CD11c⁺ cells (Supplementary Fig. S1). Subsequently, primary AMs were exposed to LPS (1 μg/ml) for 24 h in the presence or absence of graded concentrations of norepinephrine (0.1, 1, and 10 μM). ELISA analysis of cell culture supernatants revealed that LPS markedly elevated TNF-α (Fig. 1B) and IL-6 (Fig. 1C) secretion, while norepinephrine co-treatment suppressed the production of both cytokines (Fig. 1B, C). Macrophages are well established to adopt a pro-inflammatory M1 phenotype in response to inflammatory stimuli, characterized by the production of TNF-α and IL-6 [28]. Western blot analysis showed that the LPS group exhibited increased CD86 and decreased CD206 expression, whereas norepinephrine co-treatment reversed this phenotype in a dose-dependent manner, indicating a transition in macrophage polarization from the M1 pro-inflammatory subtype to the M2 anti-inflammatory phenotype (Fig. 1D–F). Similarly, flow cytometric analysis showed that LPS markedly increased CD86 mean fluorescence intensity (MFI) within F4/80^+^ AMs, while norepinephrine treatment (1 μM) significantly attenuated this increase (Fig. 1G, H). Fluorescence microscopy of DCFH-DA staining revealed strong ROS signals in the LPS group, which were markedly attenuated by norepinephrine treatment, particularly at 1 μM (Fig. 1I). Quantitative analysis confirmed a similar trend, with norepinephrine reducing ROS levels in a dose-dependent manner (Fig. 1J). Based on the above findings and considering the potential adverse effects of higher concentrations, norepinephrine was used at 1 μM in subsequent experiments. Taken together, these results suggest that norepinephrine reduces LPS-induced inflammatory responses, oxidative stress, and polarization changes in AMs in vitro.

### Norepinephrine attenuates LPS-induced ferroptosis in a dose-dependent manner

**Fig. 2:**
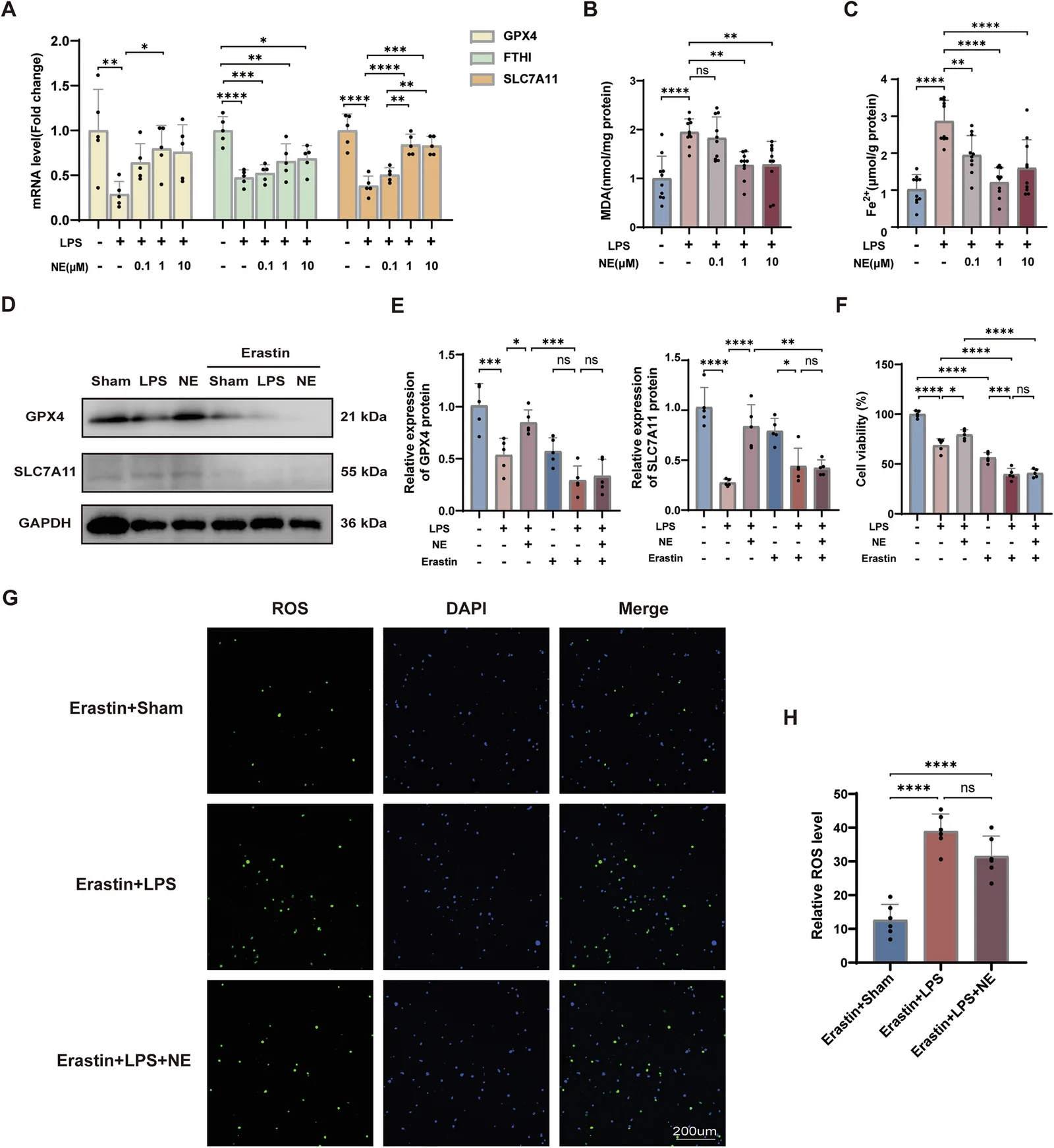
Norepinephrine attenuates LPS-induced ferroptosis in a dose-dependent manner. **A** The mRNA expression of GPX4, FTH1, and SLC7A11 was measured via quantitative real-time PCR in AMs (*n* = 5). **B** MDA levels in AMs (*n* = 10). **C** Intracellular Fe²⁺ levels in AMs (*n* = 10). **D** Representative Western blotting images of GPX4, SLC7A11 and GAPDH with or without Erastin treatment in AMs. **E** Relative protein expression levels of GPX4 and SLC7A11 to GAPDH (*n* = 5). **F** Viability of AMs was assessed by the CCK-8 assay in the presence or absence of LPS, Erastin and NE (*n* = 5). **G** Representative images of fluorescence probe for ROS in AMs. Scale bar: 200 μm. **H** Quantification of intracellular ROS fluorescence intensity in AMs (*n* = 6). Data are shown as mean ± SD. One-way ANOVA and Two-way ANOVA with Tukey’s post hoc test were used for analysis. ns not significant; *p < 0.05; **p < 0.01; ***p < 0.001; ****p < 0.0001.

To elucidate the mechanism by which norepinephrine mitigates LPS-induced macrophage inflammation, we noted that recent studies have identified ferroptosis as a critical link between macrophage polarization and proinflammatory cytokine production [29, 30]. Ferroptosis, a recently identified type of regulated cell demise, involves excessive iron buildup and oxidative lipid damage, and is modulated by critical regulators including GPX4, FTH1, and SLC7A11. Supplementary data further support this finding, demonstrating that LPS alone led to marked downregulation of these genes, accompanied by increased intracellular MDA and Fe^2+^ levels (Supplementary Fig. S2). As shown in Fig. 2A, LPS stimulation significantly downregulated the mRNA expression of ferroptosis-related genes, while norepinephrine treatment effectively reversed these changes. Both malondialdehyde (MDA) content and intracellular Fe^2+^ levels in AMs were also elevated following LPS stimulation and progressively reduced by norepinephrine treatment (Fig. 2B, C). Western blot images and densitometric analysis demonstrated that norepinephrine significantly upregulated the expression of GPX4 and SLC7A11 following LPS exposure, whereas co-administration of the ferroptosis inducer Erastin (5 μM) reversed these effects (Fig. 2D, E). In addition, CCK-8 analysis was performed to assess AM viability under the indicated conditions, showing that norepinephrine exerted a protective effect on cell viability, which was attenuated by Erastin co-treatment (Fig. 2F). ROS staining was then conducted to examine oxidative stress. In these analyses, DCFH-DA fluorescence was quantified as the mean intracellular intensity per field and expressed relative to the Sham group to allow direct comparison among all treatment conditions (Fig. 2G, H). Fluorescence remained low in the Erastin+Sham group but was markedly elevated in the Erastin+LPS group. Notably, norepinephrine co-treatment (Erastin+LPS + NE) did not effectively reduce ROS accumulation, suggesting that the antioxidative effect of norepinephrine was attenuated in the presence of Erastin (Fig. 2G). Quantitative analysis confirmed this observation (Fig. 2H). Collectively, these findings highlight ferroptosis as a key pathogenic mechanism in LPS-induced macrophage injury and support the protective effect of norepinephrine.

### Norepinephrine suppresses ferroptosis via β2-adrenergic receptor signaling

**Fig. 3:**
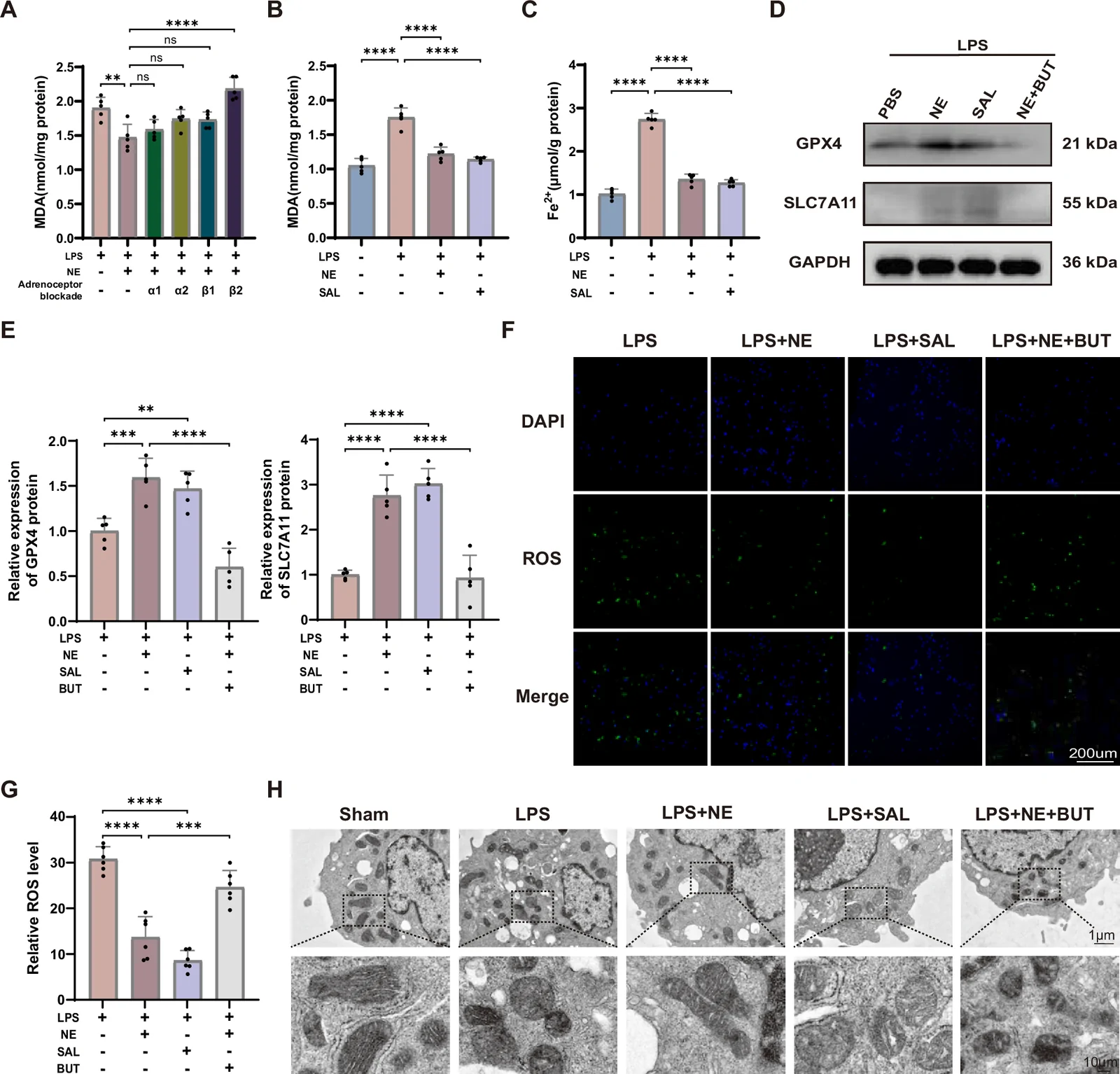
Norepinephrine suppresses ferroptosis via β2-adrenergic receptor signaling. **A** MDA levels in AMs co-treated with LPS and selective adrenergic receptor antagonists (α1, α2, β1, β2) (*n* = 5). **B, C** Intracellular MDA and Fe²⁺ levels in AMs treated with LPS and salbutamol (SAL) (*n* = 5). **D** Representative Western blotting images of GPX4, SLC7A11 and GAPDH. **E** Relative protein expression levels of GPX4 and SLC7A11 to GAPDH (*n* = 5). **F, G** Representative fluorescence images and corresponding quantification of intracellular ROS levels in AMs (*n* = 6). Scale bar: 200 μm. **H** Representative TEM images showing mitochondrial ultrastructure in AMs. Magnifications highlight mitochondrial morphology. Scale bars: upper panel, 1.0 μm; lower panel, 10.0 μm. Data are shown as mean ± SD. Two-way ANOVA with Tukey’s post hoc test was used for analysis. ns, not significant; *p < 0.05; **p < 0.01; ***p < 0.001; ****p < 0.0001.

It has been discovered that norepinephrine exerts its biological effects mainly through α- and β-adrenergic receptors, including α1, α2, β1, β2, and β3 subtypes [31]. To determine the receptor subtype involved in the antiferroptotic effect of norepinephrine, selective antagonists for α1 (prazosin, 5 μM), α2 (yohimbine, 10 μM), β1 (atenolol, 5 μM), and β2 (butoxamine, BUT, 10 μM) receptors were applied at the beginning of LPS stimulation. Among them, only butoxamine reversed the norepinephrine-mediated reduction in MDA levels, indicating a β2-AR-dependent mechanism (Fig. 3A). Based on this finding, the selective β2 agonist salbutamol (5 μM) was utilized. Both norepinephrine and salbutamol significantly reduced MDA and Fe²⁺ levels in LPS-treated cells (Fig. 3B, C). Western blot analysis showed increased expression of GPX4 and SLC7A11 after norepinephrine or salbutamol treatment, which was blocked by butoxamine (Fig. 3D, E). Consistent with these findings, immunofluorescence staining revealed that ROS accumulation was significantly reduced by norepinephrine and salbutamol, whereas this effect was abolished by co-treatment with butoxamine (Fig. 3F). Quantitative analysis of ROS intensity further confirmed these observations (Fig. 3G). In addition, salbutamol and butoxamine were also shown to modulate inflammatory cytokine levels, including TNF-α and IL-6 (Supplementary Fig. S3).

Transmission electron microscopy revealed that LPS induced mitochondrial shrinkage, membrane density, and cristae loss - hallmarks of ferroptosis. These alterations were alleviated by norepinephrine and salbutamol, but reappeared in the presence of butoxamine, confirming that β2 receptor activation protects against ferroptotic mitochondrial damage in AMs (Fig. 3H). Together, these results indicate that the anti-ferroptotic effect of norepinephrine in LPS-induced macrophage injury is specifically mediated through the β2-AR. The application of salbutamol resulted in similar anti-inflammatory and antioxidative outcomes as norepinephrine.

### Norepinephrine upregulates HDC expression and enriches oxidative stress-related pathways

**Fig. 4:**
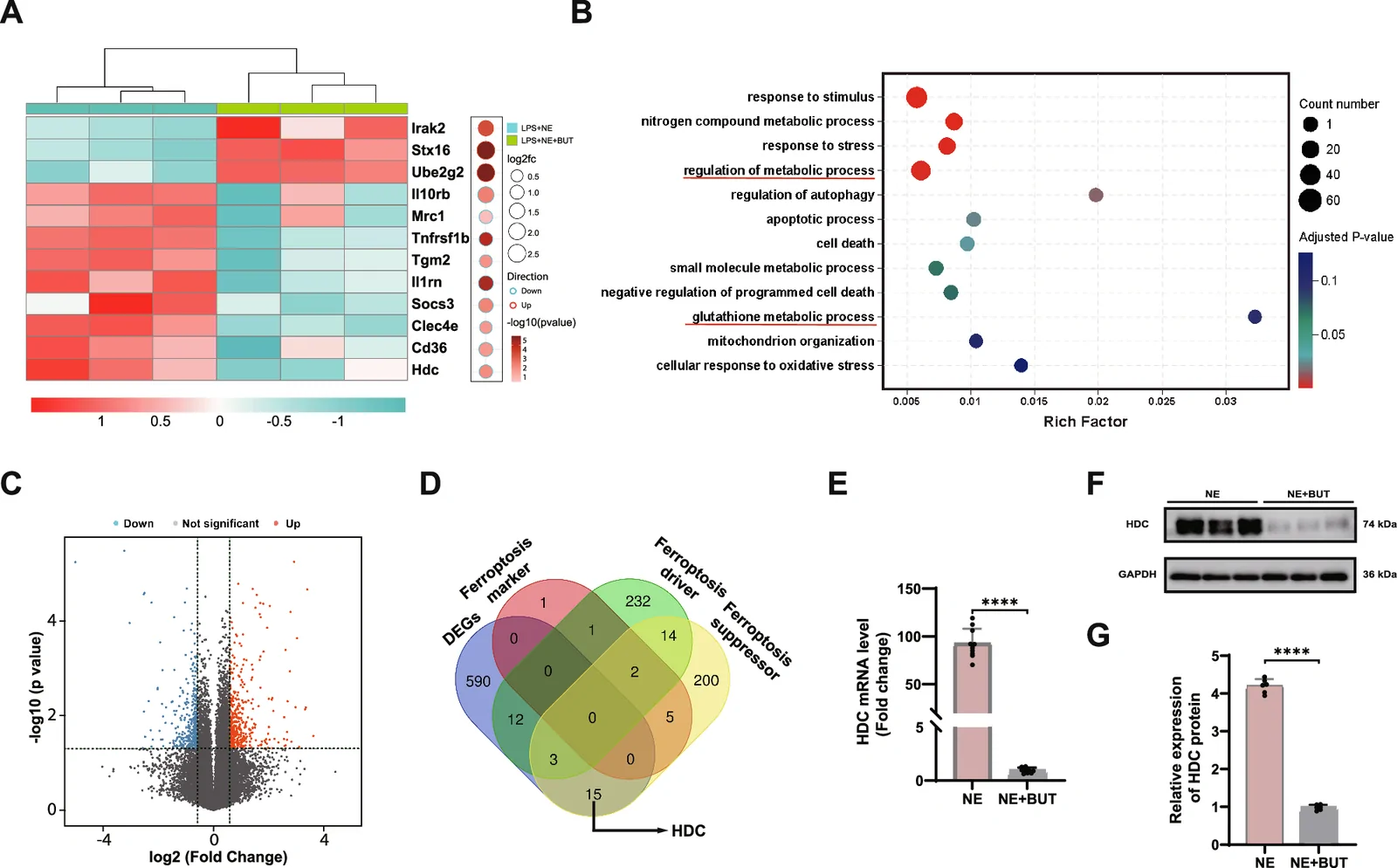
Norepinephrine upregulates HDC and activates oxidative stress-related pathways in AMs. **A** Hierarchical clustering heatmap of differentially expressed genes in AMs treated with LPS or LPS + NE (*n* = 3). **B** KEGG enrichment analyses indicating enrichment of oxidative stress response pathways. **C** Volcano plot highlighting upregulation of HDC after NE treatment. **D** Venn diagram of differentially expressed genes (DEGs) with known ferroptosis driver, marker, and suppressor gene sets. **E** The mRNA expression of HDC was measured via quantitative real-time PCR in AMs (*n* = 10). **F, G** Immunoblotting and densitometry confirming increased HDC protein levels (*n* = 6). Data are shown as mean ± SD. Two-way ANOVA with Tukey’s post hoc test was used for analysis. ns, not significant; *p < 0.05; **p < 0.01; ***p < 0.001; ****p < 0.0001.

To investigate how norepinephrine confers protection at the molecular level, transcriptomic analysis was performed in LPS-injured AMs with norepinephrine treatment. Hierarchical clustering of differentially expressed genes revealed a clear separation between the LPS + NE and LPS + NE + BUT groups, indicating distinct gene expression profiles (Fig. 4A). Bubble plot analysis demonstrated that norepinephrine-regulated genes were significantly enriched in pathway such as regulation of oxidative stress, glutathione metabolic process, and mitochondrial organization (Fig. 4B). Volcano plot analysis identified numerous differentially expressed genes (Fig. 4C). To identify ferroptosis-related targets potentially involved in the anti-inflammatory effects of norepinephrine, we integrated differentially expressed genes (DEGs) from RNA-seq data with the ferroptosis-associated gene sets (drivers, suppressors, and markers) curated from FerrDb (http://www.zhounan.org/ferrdb/current/) and relevant literature (Fig. 4D). A total of 15 genes overlapped between DEGs and ferroptosis suppressors, among which histidine decarboxylase (HDC) was selected for further validation based on its potential regulatory role in macrophage redox balance. We focused on HDC, given its reported role in redox homeostasis and ferroptosis regulation [32]. qPCR analysis was performed to confirm the upregulation of HDC mRNA, and showed a significant decrease in the NE + BUT group compared to NE alone (Fig. 4E). Western blot analysis also showed a pronounced elevation of HDC protein in norepinephrine-treated cells (Fig. 4F, G).

### Norepinephrine inhibits ferroptosis via the HDC/SLC7A11 axis in AMs

**Fig. 5:**
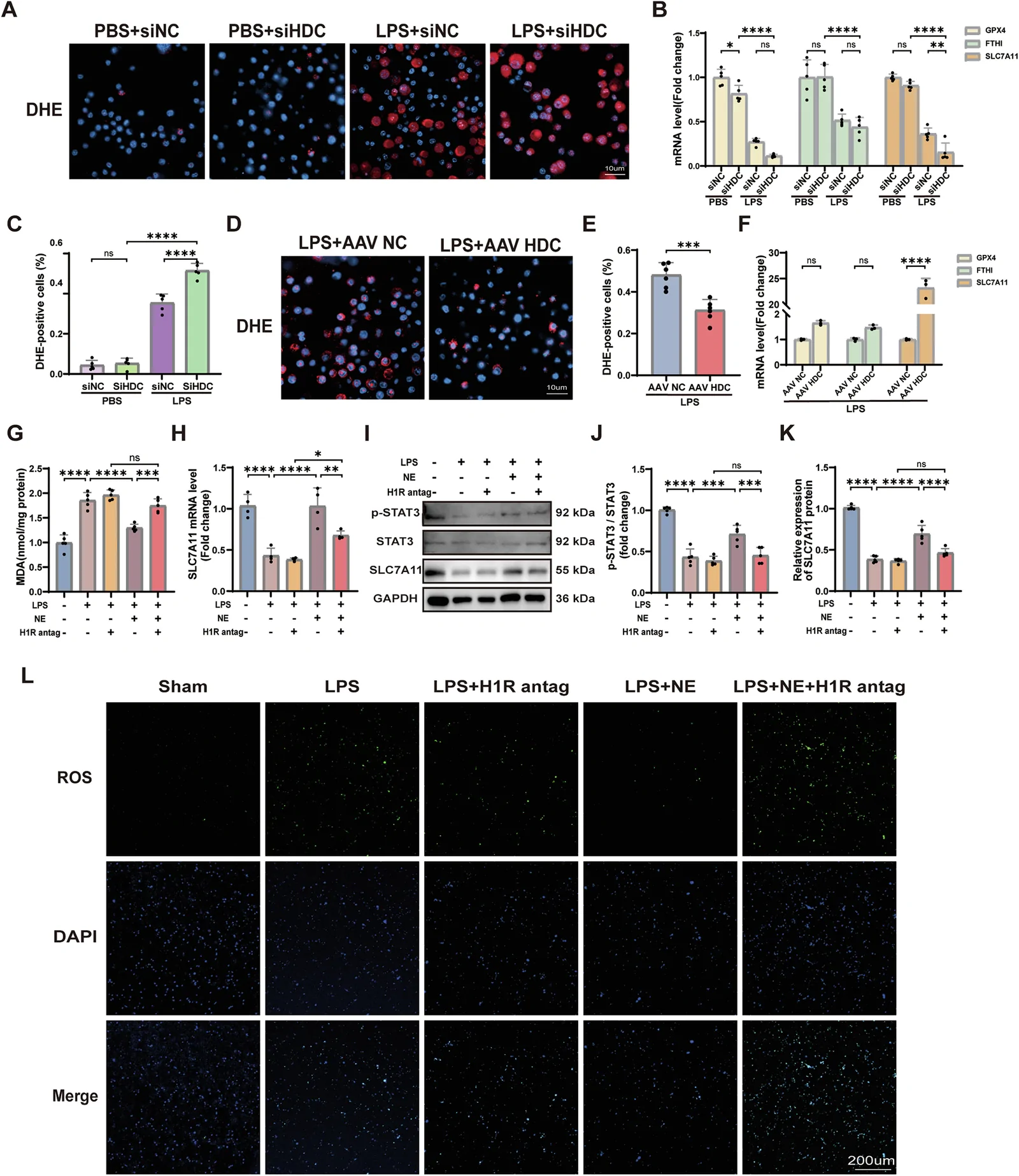
Norepinephrine inhibits ferroptosis via the HDC/SLC7A11 axis in AMs. **A** Representative fluorescence images of intracellular ROS in AMs transfected with siHDC and stimulated with LPS. Scale bar: 10 μm. **B** The mRNA expression of GPX4, FTH1, and SLC7A11 was measured via qPCR in AMs transfected with siHDC and stimulated with LPS (*n* = 5). **C** Quantification of DHE-positive cells in each group (*n* = 5). **D, E** Representative ROS fluorescence images and quantification of DHE-positive primary AMs from LPS-treated mice transduced with AAV-HDC or AAV-NC (*n* = 6). Scale bar: 10 μm. **F** The mRNA levels of GPX4, FTH1, and SLC7A11 in primary AMs (*n* = 3). **G** MDA levels in AMs (*n* = 5). **H** SLC7A11 mRNA expression in cells (*n* = 4). **I** Representative Western blotting images of p-STAT3, STAT3, SLC7A11 and GAPDH. **J** The protein expression of p-STAT3 relative to total STAT3 (*n* = 5). **K** The protein expression of SLC7A11 relative to GAPDH (*n* = 5). **L** Representative fluorescence images of ROS in AMs. Scale bar: 200 μm. Data are shown as mean ± SD. Two-way ANOVA with Tukey’s post hoc test was used for analysis. ns, not significant; *p < 0.05; **p < 0.01; ***p < 0.001; ****p < 0.0001.

Given the regulatory effect of norepinephrine on HDC expression via β2-adrenergic signaling, we next explored whether HDC plays a functional role in modulating ferroptosis and inflammation in AMs. Firstly, we knocked down the HDC gene in AMs using siRNA transfection. DHE staining demonstrated a pronounced elevation of ROS levels in the LPS+siHDC group relative to the LPS+siNC group (Fig. 5A). qPCR analysis further revealed that HDC silencing significantly downregulated SLC7A11 mRNA expression in response to LPS stimulation, whereas GPX4 and FTH1 showed a decreasing trend but did not reach statistical significance (Fig. 5B). Quantitative assessment further verified an increased proportion of DHE-positive cells following HDC silencing (Fig. 5C).

To validate these findings in vivo, rAAV-F4/80-mHDC-P2A-EGFP (5×10^12^ vg/mL, 40 μL) was administered via intratracheal injection 21 days prior to modeling to induce HDC overexpression in AMs. After 21 days, LPS (5 mg/kg) was administered intranasally to induce ARDS. Twenty-four hours post-induction, lung samples, BALF, and primary AMs were collected for downstream analyses. Successful in vivo transduction and HDC overexpression were confirmed by EGFP fluorescence in the alveolar region and by significantly increased HDC mRNA and protein levels in BALF-derived AMs from AAV-HDC–treated mice compared with AAV-NC controls (Supplementary Fig. S4A–C). Subsequently, HDC overexpression was found to significantly reduce ROS accumulation (Fig. 5D) and the proportion of DHE-positive cells (Fig. 5E) compared with the AAV-NC group. Consistently, the AAV-HDC group exhibited significantly increased transcript levels of SLC7A11 (Fig. 5F), supporting the notion that HDC alleviates LPS-induced ferroptosis in AMs and contributes to the cytoprotective phenotype.

It is well established that HDC catalyzes the synthesis of histamine, a biologically active monoamine involved in diverse physiological and pathological processes. Histamine mediates its biological functions via four distinct G protein-coupled receptors (GPCRs), namely H1R, H2R, H3R, and H4R. Notably, H1R has been implicated in the regulation of ferroptosis, suggesting a mechanistic link between histamine signaling and redox imbalance–associated cell death [33]. HDC deficiency has also been reported to promote ferroptosis in cardiomyocytes via inhibition of the H1R-STAT3-SLC7A11 pathway [32]. To investigate this mechanism, AMs were exposed to LPS and norepinephrine, with parallel groups receiving an H1R antagonist to assess receptor-specific effects. Our results demonstrated that although norepinephrine treatment reduced MDA content, this effect was abolished by H1R blockade, indicating that H1R signaling is required for norepinephrine-mediated suppression of lipid peroxidation (Fig. 5G). Similarly, SLC7A11 mRNA expression, which was suppressed by LPS, was restored following norepinephrine treatment, but this restoration was prevented by H1R antagonist (Fig. 5H). Western blot analysis showed that the upregulation of phosphorylated STAT3 (p-STAT3) and SLC7A11 proteins by norepinephrine was significantly inhibited by either H1R antagonist (Fig. 5I) or the STAT3 inhibitor c188-9 (Supplementary Fig. S5). Quantitative analysis of SLC7A11 and p-STAT3 protein levels confirmed these findings (Fig. 5J, K). Similarly, ROS staining revealed that the decrease in intracellular ROS levels conferred by norepinephrine was completely abrogated by H1R antagonist (Fig. 5L). This finding supports the involvement of the HDC-STAT3–SLC7A11 axis as a downstream effector pathway mediating ferroptosis inhibition.

### Salbutamol alleviates lung injury via HDC-dependent ferroptosis suppression

To further verify the role of norepinephrine in alleviating ARDS through the inhibition of ferroptosis, in vivo experiments were conducted. We pretreated mice with salbutamol (10 mg/kg) 3 h before intranasal instillation of LPS (5 mg/kg) to clarify the contribution of β2-adrenergic signaling. Erastin (10 mg/kg) was administered intraperitoneally concurrently with intranasal LPS stimulation. HDC overexpression via intratracheal viral delivery was performed as previously described. Lung tissues were harvested 24 h after LPS administration for subsequent analyses.

**Fig. 6:**
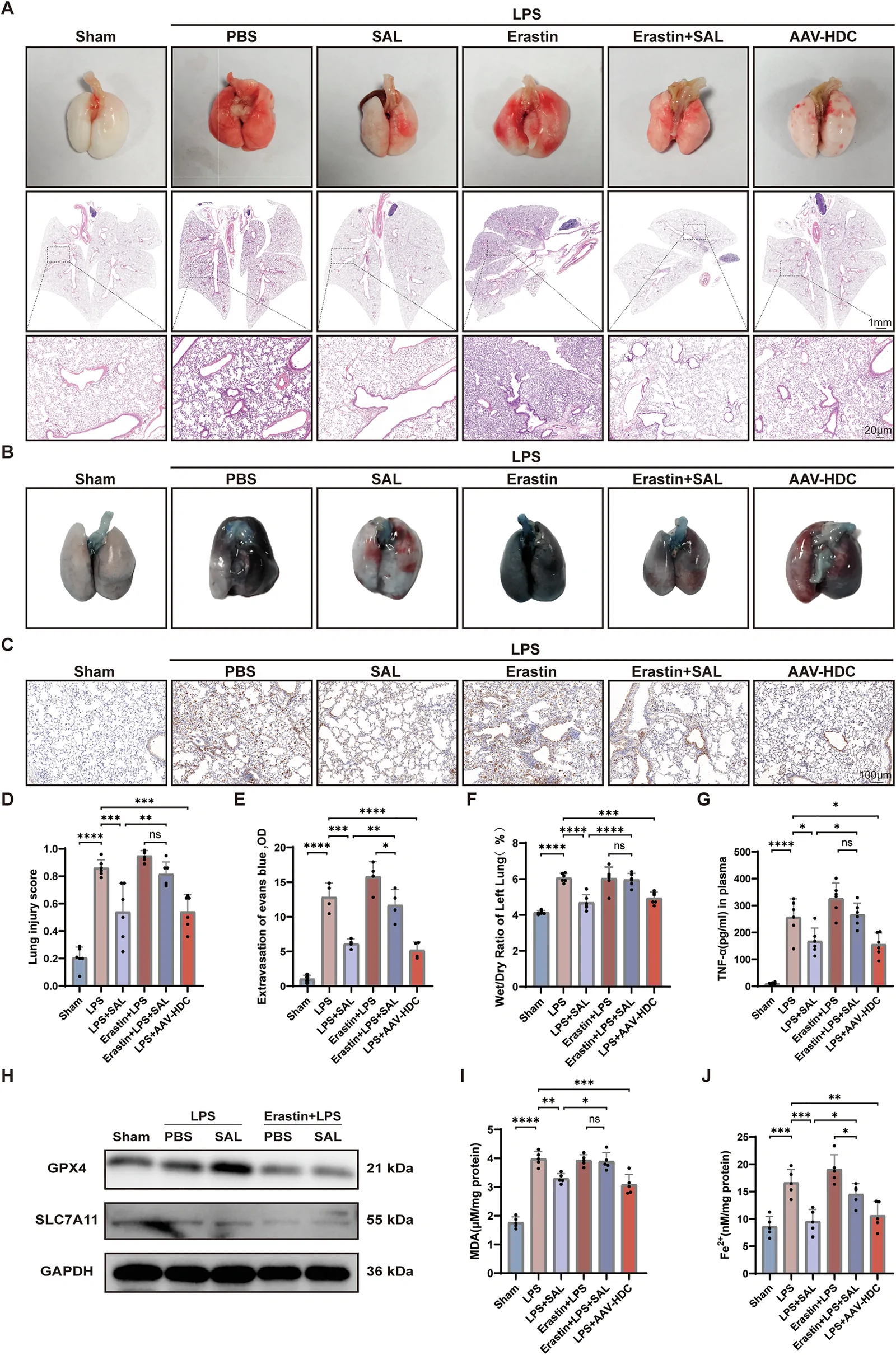
Salbutamol alleviates lung injury via HDC-dependent ferroptosis suppression. **A** Representative gross morphology and H&E staining sections of mouse lung tissues. Scale bars: 1 mm (middle panels), 20 μm (bottom panels). **B** Gross images of Evans blue-stained lungs. **C** 4-HNE expression in the lung tissues of mice was tested by immunohistochemistry. Scale bar: 100 μm. **D** Lung injury scores based on histological evaluation (*n* = 6). **E** Quantification of Evans blue extravasation in lung tissue (*n* = 4). **F** Lung wet/dry weight ratio (W/D) (*n* = 6). **G** Plasma TNF-α levels measured by ELISA (*n* = 6). **H** Representative Western blotting images of GPX4 and SLC7A11 in lung tissue. **I** MDA content in lung tissue (*n* = 5). **J** Fe²⁺ levels in lung tissue (*n* = 5). Data are shown as mean ± SD. Two-way ANOVA with Tukey’s post hoc test was used for analysis. ns, not significant; *p < 0.05; **p < 0.01; ***p < 0.001; ****p < 0.0001.

Histological analysis revealed that LPS induced severe lung injury, evidenced by hemorrhage, edema, and alveolar destruction. Both salbutamol treatment and alveolar macrophage-targeted HDC overexpression significantly reduced lung injury scores, while the protective effect of salbutamol was partially reversed by Erastin (Fig. 6A, D). Similarly, pulmonary vascular permeability and Evans blue extravasation were decreased in salbutamol and AAV-HDC groups (Fig. 6B, E). Lung W/D ratios (Fig. 6F) and TNF-α levels (Fig. 6G) were also reduced by salbutamol or HDC overexpression, with Erastin attenuating these effects. 4-HNE staining confirmed reduced lipid peroxidation in treated groups (Fig. 6C). At the molecular level, western blot analysis showed that salbutamol increased GPX4 and SLC7A11 expression in lung tissue (Fig. 6H), which was further supported by densitometric quantification normalized to GAPDH (Supplementary Fig. S6A, B). In parallel, salbutamol decreased MDA content and Fe²⁺ accumulation, and these ferroptosis-associated changes were further enhanced by HDC overexpression (Fig. 6I, J). These results suggest that salbutamol mitigates LPS-triggered pulmonary damage by suppressing ferroptosis in AMs. Notably, targeted HDC overexpression in AMs conferred comparable protective effects, and our in vitro data further support a potential link between β2-adrenergic signaling and HDC expression.

## Discussion

**Fig. 7:**
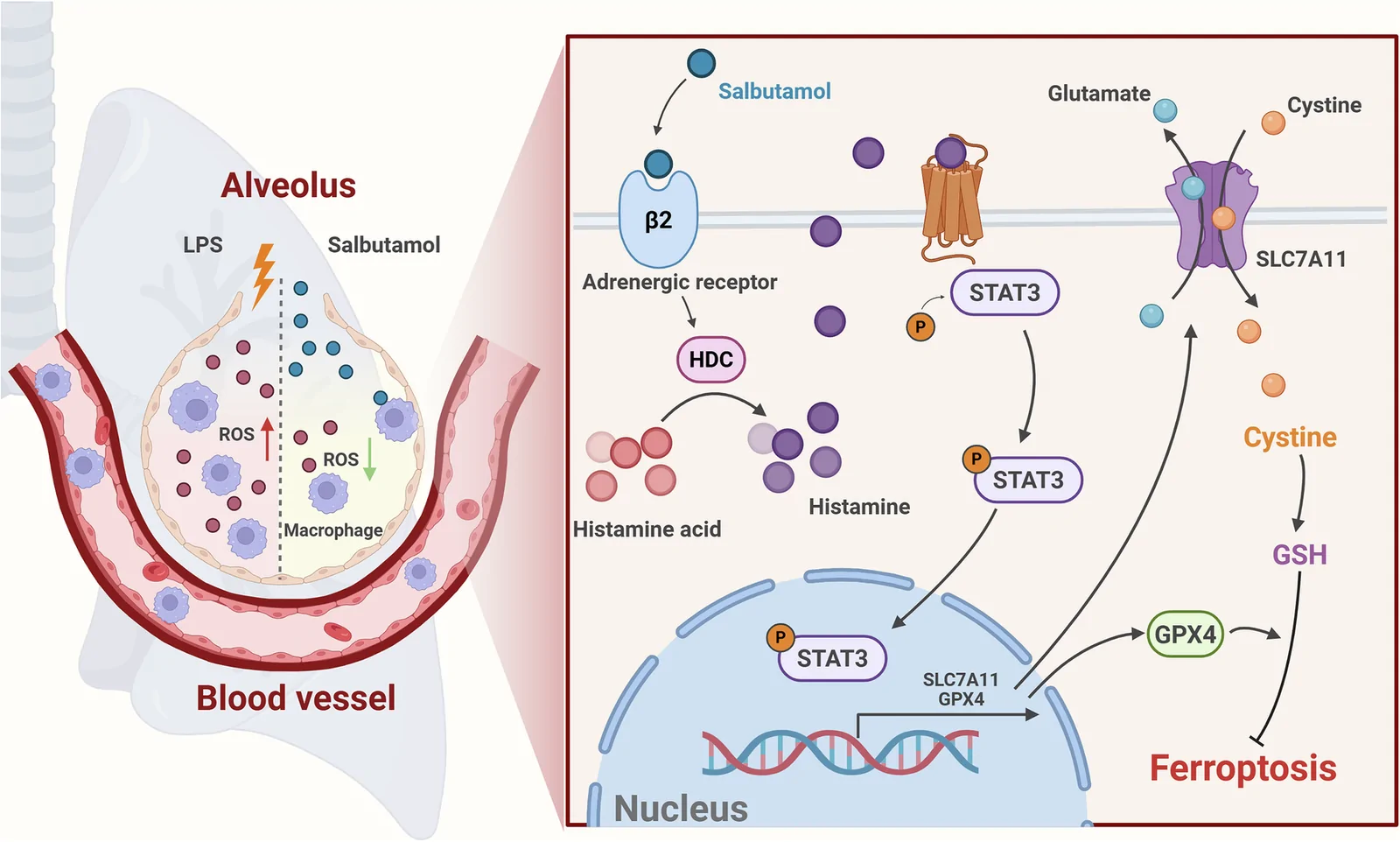
Activation of β2-adrenergic signaling upregulates HDC to inhibit ferroptosis and relieve ARDS. Schematic illustration of β2-adrenergic signaling-mediated protection by salbutamol against LPS-induced ferroptosis via the HDC/SLC7A11 pathway.

Although neuroimmune regulation is increasingly recognized as a critical modulator of inflammation, the precise role of sympathetic activity in acute inflammatory conditions, including ARDS, remains poorly defined. In this study, we demonstrated that norepinephrine signaling alleviates LPS-induced acute lung injury by targeting AMs and modulating ferroptosis via the HDC/SLC7A11 axis. We found that sympathetic activation, specifically β2-AR signaling, upregulated HDC expression in AMs, thereby enhancing endogenous histamine production. Through both in vivo and in vitro models of ARDS, we showed that histamine/H1R signaling mitigated oxidative stress and ferroptotic cell death in macrophages, ultimately preserving lung function. Moreover, blockade of β2-AR or H1R exacerbated macrophage ferroptosis and lung injury, underscoring the specificity of this axis. Our findings reveal a previously unrecognized neuroimmune regulatory mechanism providing new insight into the resolution of inflammation and redox imbalance in ARDS (Fig. 7).

ARDS is a life-threatening pulmonary condition marked by pronounced pulmonary inflammation, disruption of the alveolar-capillary barrier, and extensive recruitment of neutrophils and macrophages into the lung interstitium and bronchoalveolar compartments [34]. Emerging evidence implicates ferroptosis-an iron-dependent, lipid peroxidation-driven form of regulated cell death-as a pivotal mechanism underlying the pathogenesis of multiple inflammatory disorders [35, 36]. Notably, the inflamed alveolar microenvironment in ARDS is often accompanied by disrupted iron handling and excessive oxidative stress, which collectively create an iron-overload niche favoring lipid peroxidation and ferroptotic vulnerability [37]. As the dominant resident immune population within the bronchoalveolar compartment, AMs play a central role in iron scavenging and buffering oxidative stress [38]. Studies have shown that macrophages can resist ferroptosis under conditions of heme/iron overload through HO-1/FTH induction and ferroportin-mediated iron export, while also phagocytosing and clearing ferroptotic cells [39]. However, sustained iron loading and excessive oxidative stress may drive a pro-inflammatory activation program in AMs, and ferroptosis is typically triggered when lipid peroxidation exceeds antioxidant defense capacity under iron-overload conditions [29]. In this context, AM polarization programs are increasingly appreciated to be coupled to ferroptotic susceptibility, and M2 macrophages have been reported to exhibit greater sensitivity to ferroptosis than M1 macrophages [40], as exemplified in other inflammatory settings where preferential ferroptosis of M2-polarized macrophages disrupts the M1/M2 balance [41]. Importantly, ferroptosis of AMs is increasingly recognized as an active driver of ARDS progression by amplifying oxidative stress and propagating inflammatory injury [42, 43]. Recent findings have shown that melatonin suppresses macrophage ferroptosis by inhibiting NCOA4-mediated ferritinophagy, thereby reducing lipid peroxidation and improving outcomes in septic ARDS models [44]. However, the regulatory networks governing macrophage ferroptosis in ARDS remain poorly defined. Modulating ferroptotic pathways in AMs holds potential as a novel intervention approach for ARDS management [27].

Norepinephrine, a key catecholamine released from sympathetic nerve terminals, regulates cardiovascular and metabolic homeostasis and exerts context-dependent immunomodulatory effects shaped by distinct adrenergic receptor subtypes (α1, α2, β1, and β2) [45]. Notably, β2-AR signaling has been revealed as a dominant anti-inflammatory pathway across various immune cell types. A recent study has shown that activation of hypothalamic paraventricular nucleus (PVN) corticotropin-releasing hormone (CRH) neurons restrains neutrophil-driven lung injury through a norepinephrine–β2-AR signaling cascade [46]. Studies on neuroinflammation and neuropathic pain have shown that norepinephrine attenuates inflammatory responses by inhibiting microglial activation [47]. It has also been reported that activation of sympathetic signaling in macrophages promotes anti-inflammatory phenotypic shift, partially by upregulating Tim3, which leads to diminished systemic inflammation and enhanced protection against tissue injury [48]. Emerging studies indicate that neuroimmune regulation can alleviate ARDS by suppressing ferroptosis, as demonstrated by electroacupuncture acting through α7 nicotinic acetylcholine receptors [49]. However, the specific relationship between sympathetic neuroimmune regulation, ferroptosis, and ARDS has not yet been fully elucidated. Our study further demonstrated that norepinephrine suppresses ferroptosis in LPS-induced macrophage injury specifically through β2-AR signaling. Pharmacological activation of the β2-AR with salbutamol reproduced the protective effects of norepinephrine, while selective blockade with butoxamine abolished these benefits, as evidenced by reductions in MDA, Fe^2+^, and ROS levels. In our in vitro assays, intracellular ROS was assessed using DCFH-DA and DHE, two widely applied fluorescence probes that primarily report overall cellular ROS and are not fully specific for lipid peroxides, which are more directly linked to ferroptosis [50]. Therefore, our interpretation of ferroptosis relies on a combination of readouts, including lipid peroxidation markers (MDA and 4-HNE), iron accumulation, and canonical ferroptosis regulators such as GPX4 and SLC7A11. Notably, salbutamol treatment also upregulated the expression of SLC7A11, FTH1, and GPX4, and preserved mitochondrial ultrastructure, indicating a suppression of ferroptotic cell death. These results align with previous work by Liyan Hou and colleagues, who showed that norepinephrine deficiency aggravates ferroptosis, whereas normal norepinephrine levels help maintain antioxidant defenses, preserve mitochondrial function, and sustain intracellular homeostasis, thereby suppressing neuronal ferroptosis in mouse parkinson’s disease model [26]. Meanwhile, inhibition of macrophage ferroptosis via the Nrf2 signaling pathway has been demonstrated to alleviate ARDS [51]. Taken together, these mechanistic insights not only reinforce the neuroimmune axis as a key modulator of ferroptosis in the context of acute lung injury, but also suggest that targeting sympathetic β2-adrenergic signaling may offer a promising therapeutic avenue for the management of ARDS. Importantly, salbutamol-a clinically approved selective β2-AR agonist widely used in clinical practice-exhibited protective effects comparable to those of norepinephrine in our model. Moreover, a previous in vitro study showed that the salbutamol directly inhibited TNF-α production by AMs [52]. These findings suggest that targeting macrophage ferroptosis via β2-AR signaling may represent a viable strategy for mitigating inflammation-induced lung injury. Given its well-characterized safety profile and extensive clinical application, our results provide a rational basis for repurposing β2-AR agonists as immunomodulatory therapeutics in ARDS.

HDC catalyzes the decarboxylation of histidine to generate histamine, a biogenic amine that functions as a neurotransmitter and modulator of various metabolic processes. Depending on its receptor context, histamine can exert either pro-inflammatory or anti-inflammatory effects [31, 53]. Previous studies have shown that histamine deficiency in HDC knockout (HDC⁻/⁻) mice significantly reduces blood perfusion and impairs muscle regeneration [54]. In addition, histamine attenuates cardiomyocyte autophagy and apoptosis in acute myocardial infarction via H1 receptor signaling [55]. Our enrichment analysis indicated that norepinephrine-induced upregulation of HDC is associated with ferroptosis-related antioxidant and metabolic pathways, suggesting an indirect regulatory role in ferroptosis susceptibility. Similarly, recent researches have also suggested a protective role for HDC against ferroptosis. For example, in a cisplatin-induced ototoxicity model, histamine deficiency resulting from HDC knockout was shown to exacerbate ferroptotic cell death in cochlear hair cells, as evidenced by increased lipid peroxidation, iron accumulation, and decreased antioxidant capacity [56]. Studies in myocardial infarction models have demonstrated that HDC deficiency aggravates cardiac injury, characterized by increased neutrophil infiltration, elevated ROS production, and excessive formation of neutrophil extracellular traps (NETs) [32]. Multi-omics analyses suggest that HDC and its metabolic pathways may contribute to resistance against ferroptosis by enhancing cellular antioxidant defenses in pressure-overload cardiac dysfunction [57]. In the context of doxorubicin-induced cardiotoxicity, disruption of histamine signaling—either by HDC deletion or H1 receptor inhibition—leads to increased susceptibility to ferroptosis [58]. Mechanistically, the HDC/histamine-H1R axis protects against ferroptosis by activating the STAT3-SLC7A11 pathway, which promotes glutathione biosynthesis and enhances cellular antioxidant defenses [32]. Consistent with these observations, in our ARDS model intratracheal delivery of a F4/80 promoter–driven AAV-HDC-P2A-EGFP construct resulted in robust HDC overexpression in lung AMs, as evidenced by EGFP staining in alveolar regions and by increased HDC mRNA and protein levels in BALF-derived AMs (Supplementary Fig. S4). Moreover, our experiments showed that the anti-ferroptotic effect of norepinephrine was partially attenuated by both an H1 receptor antagonist and the STAT3 inhibitor C188-9, further confirming that the HDC/histamine-H1R/STAT3 axis is essential for mediating the protective actions of norepinephrine against ferroptosis. These results highlight the importance of HDC in maintaining cellular redox balance, further confirming the crucial role of the HDC/SLC7A11 signaling cascade in macrophage ferroptosis regulation.

However, several limitations should be noted. Although primary AMs were used, they may not fully reflect the complexity of human AMs or the in vivo immune microenvironment. Our study primarily focused on macrophages, while the involvement of other immune and structural cells in ARDS remains unexplored. Moreover, the neuroimmune mechanisms linking β2-AR signaling to macrophage function, such as the upstream regulation of HDC and its downstream interactions, require further investigation. Future studies should also assess the therapeutic value of β2-agonists like salbutamol in broader inflammatory contexts.

## Conclusion

Our study identifies a novel neuroimmune mechanism in which norepinephrine suppresses macrophage ferroptosis to alleviate LPS-induced ARDS. This effect is mediated by β2-AR activation, which promotes histamine synthesis and triggers downstream signaling via the HDC/SLC7A11 axis. These findings highlight a previously unrecognized link between sympathetic signaling and ferroptosis regulation, offering new insights into the immunopathogenesis of ARDS and a potential therapeutic strategy targeting neuroimmune interactions.

## Materials and methods

### Mouse model

Male C57BL/6 mice (6-10 weeks old, 20–25 g; GemPharmatech, Guangzhou, China) were maintained under specific-pathogen-free (SPF) conditions in individually ventilated cages, with unrestricted access to standard chow and water. After a 1-week acclimation period, animals were randomly allocated to either sham or ARDS groups.

To establish the ARDS model, mice received an intranasal instillation of lipopolysaccharide (LPS; 5 mg/kg, L2880, Sigma-Aldrich, USA) dissolved in 50 μL sterile saline. Mice in the sham group were administered an equal volume of sterile saline. For intervention studies, salbutamol (SAL; 10 mg/kg, T1139, TargetMol, USA) was administered by nebulization 3 h prior to LPS exposure. Nebulized saline was administered to control mice following the same schedule. At 24 h after LPS exposure, mice were euthanized by CO₂ inhalation under anesthesia. Blood and bronchoalveolar lavage fluid (BALF) samples were collected. Lung tissues were either fixed in 4% neutral-buffered formalin or rapidly frozen in liquid nitrogen and stored at −80 °C for downstream analysis.

All experimental procedures were approved by the Institutional Animal Care and Use Committee (IACUC) of Jennio Bio, with ethical approval number #JENNIO-IACUC-2025-A015.

### Isolation, culture and treatment of alveolar macrophages

AMs were isolated from C57BL/6 mice via bronchoalveolar lavage (BAL). Lavage was performed using pre-warmed PBS containing 2 mM EDTA and 0.5% fetal bovine serum (FBS, S711-001S, lonsera, China), with 10 sequential instillations of 1 mL buffer through a tracheal cannula per mouse. BALF samples were passed through a 70 μm strainer and centrifuged at 300×g for 5 min at 4 °C. Red blood cells were lysed with hemolysis buffer, and viable cells were counted using Trypan Blue exclusion. For culture, 3-4×10⁵ AMs were seeded into non-treated 6-well plates containing RPMI-1640 (RPMI 1640, GIBCO, USA) supplemented with 10% FBS and 1% penicillin-streptomycin (0503, ScienCell, USA). Cells were incubated at 37 °C in a humidified atmosphere with 5% CO₂. After incubation at 37 °C for 4 h to allow adherence, non-adherent cells (mainly neutrophils and other leukocytes) were removed by washing three times with pre-warmed PBS, leaving a purified adherent AM population (>95%) [59]. The purified AMs were then cultured in fresh complete medium for an additional 20 h (total culture time of 24 h after isolation) before pharmacological treatments and LPS stimulation were initiated. AM identity and purity were validated by cytospin staining, F4/80 immunofluorescence of adherent cells, and flow-cytometric analysis of Siglec-F and CD11c expression in BALF-derived cells, confirming an AM purity consistently >90% in ARDS groups (Supplementary Fig. S1).

After this equilibration period, AMs were exposed to LPS (1 μg/mL) for 24 h. Norepinephrine (0.1 μM, 1 μM, or 10 μM; HY-13715, MCE, China), salbutamol (100 nM; T1139, TargetMol, USA), and butoxamine (10 μM; HY-118470, MCE, China) were administered 3 h before LPS stimulation. Erastin (5 μM; 571203-78-6, GLPBIO, USA) was co-administered with LPS to induce ferroptosis under inflammatory conditions. All samples were collected 24 h after LPS stimulation for subsequent analyses.

### Cytospin staining

AMs were seeded onto sterile glass coverslips placed in 24-well plates and cultured under the same conditions as described above. After 24 h of adherence, the coverslips were gently washed with PBS and processed for Wright-Giemsa staining (G1020, Solarbio, China) or Diff-Quik staining (D030-1-3, jiancheng, China) strictly according to the manufacturers’ instructions. After staining, coverslips were mounted with a resin-based mounting medium and examined under a bright-field microscope (MiniCore tissue arrayer, LabChina Co., Limited, China) to assess cell morphology.

### Enzyme-linked immunosorbent assay (ELISA)

Levels of TNF-α and IL-6 in the culture supernatants of AMs were quantified using commercial ELISA kits for mouse TNF-α (MM-0132M2, MEIMIAN, China) and IL-6 (MM-0163M2, MEIMIAN, China), following the protocols provided by the manufacturer.

### Cell viability

Cell viability was assessed using a Cell Counting Kit-8 (CCK-8; Biosharp, China). Briefly, cells were seeded into 96-well plates at a density of 5×10³ cells/well and allowed to adhere overnight. After the indicated treatments, 10 μL of CCK-8 solution was added to each well containing 100 μL of culture medium, followed by incubation at 37 °C for 2 h. The absorbance at 450 nm was measured using a microplate reader (Spark 10 M; Tecan, Switzerland), and cell viability was calculated as a percentage of the absorbance of the untreated control group.

### Western blotting

Proteins were isolated from AMs using RIPA buffer (P0039, Beyotime, China), and quantified with a BCA assay kit (PC0020, Solarbio, China). Equal protein amounts (20 μg per lane) were resolved via 12% SDS–PAGE and electrotransferred onto PVDF membranes. After blocking in 3% BSA for 1 h at room temperature, membranes were incubated overnight at 4 °C with the following primary antibodies: SLC7A11 (26864-1-AP, Proteintech, 1:1000), FTH1 (ab75973, Abcam, 1:1000), GPX4 (GB124327, Servicebio, 1:1000), HDC (ab315444, Abcam, 1:1000), STAT3 (YT4443, Immunoway, 1:1000), p-STAT3 (Tyr705; YP0251, Immunoway, 1:1000), and GAPDH (10494-1-AP, Proteintech, 1:5000). After TBST washes, membranes were incubated with HRP-conjugated goat anti-rabbit IgG (H + L) (SA00001-2, Proteintech) or HRP-conjugated goat anti-mouse IgG (H + L) (SA00001-1, Proteintech) at a dilution of 1:5000 for 1 h at room temperature. Signal detection was performed using enhanced chemiluminescence substrate (PE0010, Solarbio, China), and band intensities were analyzed with ImageJ software.

### Flow cytometry analysis

For flow cytometry analysis, AMs were resuspended in staining buffer and blocked with anti-mouse CD16/32 (553142 BD, USA) at 4°C for 15 min to prevent nonspecific binding. Cells were stained with FITC-CD45 (E-AB-F1136C, Elabscience, China), APC-F4/80 (E-AB-F0995E, Elabscience, China), and PE-CD86 (E-AB-F0994UD, Elabscience, China) for 30 min at 4 °C in the dark. After staining and washing, cells were resuspended in staining buffer, and 7-AAD (5 μl, 420403, BioLegend, USA) was added immediately before flow cytometric analysis for live/dead discrimination. Data were processed using FlowJo software (version 10.10, Tree Star, USA), and CD86^+^ cells within the CD45^+^F4/80^+^ macrophage population were quantified.

BALF cells were processed for flow cytometric analysis of PE/Cyanine7-Siglec-F (AN00629H, Elabscience, China) and APC-CD11c (E-AB-F0991E, Elabscience, China) expression to assess AM purity. Surface staining, washing, and acquisition were performed as previously described [59]. Data were analyzed with FlowJo software.

### MDA and Fe^2+^ measurement

Malondialdehyde (MDA) and ferrous iron (Fe^2+^) levels in cells and tissues were assessed using a lipid peroxidation detection kit (E-BC-K025-M-96T, Elabscience, China) and a colorimetric iron quantification kit (MA0647-2, Meilunbio, China), respectively, following the protocols provided by the manufacturers.

### ROS measurement

Intracellular reactive oxygen species (ROS) levels were evaluated using a commercial detection kit (S0033S, Beyotime, China) according to the manufacturer’s instructions. Briefly, cells were incubated with 10 μM 2′,7′-dichlorodihydrofluorescein diacetate (DCFH-DA) diluted in serum-free medium and incubated at 37 °C for 20 min in the dark, washed three times with serum-free medium, and nuclei were counterstained with 4′,6-diamidino-2-phenylindole (DAPI) (28718-90-3, Solarbio, China). Fluorescence images were acquired on a confocal laser scanning microscope (Zeiss LSM880, Carl Zeiss, Germany; excitation/emission: 488/525 nm). Intracellular ROS levels were quantified in ImageJ as the mean DCFH-DA fluorescence intensity per field from 4-6 randomly selected images per sample acquired under identical settings, after background subtraction and normalization to the number of DAPI-positive nuclei; the average value of all fields was used as one data point for statistical analysis.

To assess lipid peroxidation, cells were stained with 10 μM dihydroethidium (DHE, S0064S, Beyotime, China) in the dark for 30 min and visualized via same fluorescence microscopy (excitation/emission: 518/605 nm). For quantification, DHE-positive cells were counted together with total DAPI-positive nuclei in 4-6 randomly selected fields per sample, and the percentage of DHE-positive cells among total cells was calculated; the mean value per sample was used for statistical analysis.

### siRNA transfection

AMs were seeded in 48-well plates and transfected with siHDC or a non-targeting control (siNC) using Lipofectamine 3000 (L3000015, Invitrogen, USA) according to the manufacturer’s protocol. The detailed sequences of these siRNAs are provided in Table 1 for reference. The siRNA was used at a final concentration of 50 nM. The culture medium was replaced 6 h after transfection, and cells were harvested 48 h post-transfection for downstream assays. Knockdown efficiency was verified by RT-qPCR and/or immunoblotting.

**Table 1:** Gene primer sequences.

Gene	Forward primer (5′ → 3′)	Reverse primer (5′ → 3′)
SLC7A11	CCTCTGCCAGCTGTTATTGTT	CCTGGCAAAACTGAGGAAAT
FTH1	CCATCAACCGCCAGATCAAC	GAAACATCATCTCGGTCAAA
GPX4	GCCTGGATAAGTACAGGGGTT	CATGCAGATCGACTAGCTGAG
HDC	ACTCCAAATGTGCAGCCTGGATACC	GGCTAGATGCCCACGTGAATCCTAA
STAT3	GCCGCCGTAGTGACAGAGAA	GGCAGCAACATCCCCAGAGT
GAPDH	GTGGCAAAGTGGAGATTGTTG	CGTTGAATTTGCCGTGAGTG
SiHDC	GGAAAGAGAUGGUGGAUUAUU	UAAUCCACCAUCUCUUUCCUU
SiNC	UUCUCCGAACGUGUCACGUUU	ACGUGACACGUUCGGAGAAUU

### Transmission electron microscopy (TEM)

Cells were initially fixed in 2.5% glutaraldehyde, followed by secondary fixation with 1% osmium tetroxide for 2 h at room temperature. After rinsing in phosphate buffer, samples underwent graded ethanol dehydration and acetone infiltration, then embedded in epoxy resin and polymerized overnight at 60–70 °C. Ultrathin sections (70–90 nm) were prepared in a Leica EM UC7 ultramicrotome and stained with uranyl acetate and lead citrate. The sections were then observed under a transmission electron microscope (JEM-1200EX, JEOL, Japan), and representative ultrastructural images were acquired for analysis.

### RNA isolation and real-time quantitative PCR(RT-qPCR) analysis

Total RNA was isolated from AMs by using TRIzol reagent (10296010, Thermo Fisher Scientific, USA) following standard procedures. Complementary DNA (cDNA) was generated from 1 μg of RNA with the HiScript III RT SuperMix (R323-01, Vazyme, China). Quantitative real-time PCR was performed using ChamQ Universal SYBR qPCR Master Mix (Q711-02, Vazyme, China) on a real-time detection system following standard cycling parameters. GAPDH was used as the internal reference gene for normalization. The primer information is provided in Table 1. Relative transcript levels were quantified using the 2^–ΔΔCt approach.

### AAV intratracheal administration

Mice received intratracheal rAAV-F4/80-mHDC-P2A-EGFP or vector-control rAAV-F4/80-P2A-EGFP (BolinKais Biotechnology, China), each at 5 × 10^12 vg/mL; viruses were used as supplied without further dilution. Mice were anesthetized with tribromoethanol, positioned supine, and a sterile flexible catheter (≈22 G) was advanced under direct visualization into the trachea. 40 μL viral suspension (~2.5×10^11 vg/mouse) was instilled slowly (~1 μL/s) via a microliter syringe. Successful intratracheal delivery was verified by synchronous thoracic excursions without oropharyngeal leakage. Animals were maintained head-up (30°–45°) for 1 ~ 2 min and then recovered on a warming pad. Injections were performed by an operator blinded to group allocation. In ARDS experiments, mice were challenged intranasally with LPS (5 mg/kg) on day 21 after AAV administration, and tissues were harvested 24 h after LPS for downstream analyses. For assessment of AAV transduction and HDC overexpression, lung tissues and BALF-derived AMs were also collected 21 days post-injection.

### H&E staining and lung injury scoring

Lung samples underwent 4% paraformaldehyde fixation, followed by paraffin embedding and microtome sectioning at 4 μm. Sections were deparaffinized, rehydrated through graded ethanol (95%, 85%, 75%), and stained with hematoxylin and eosin. Lung injury severity was evaluated according to five histopathological parameters: (A) neutrophilic infiltration within alveolar spaces, (B) interstitial neutrophil accumulation, (C) formation of hyaline membranes, (D) intra-alveolar proteinaceous exudate, and (E) alveolar septal thickening. The injury index was derived using the following formula: [(20×A) + (14×B) + (7×C) + (7×D) + (2×E)] divided by the number of 100× microscopic fields, yielding a continuous score ranging from 0 to 1. Two independent, blinded pathologists conducted the histological assessments.

### Evans Blue dye extravasation assay

Following anesthesia, mice received a 100 μL intravenous injection of 1% Evans Blue solution (E2129, Sigma-Aldrich, USA). After 30 min of circulation, mice were euthanized, and the pulmonary vasculature was perfused through the right ventricle with 50 mL PBS to remove intravascular dye. Lungs were collected, weighed, and incubated in 2 mL of N,N-Dimethylformamide (DMF) (N807507, Macklin, China) at 55 °C for 24 h to extract the dye. Supernatant absorbance was detected at 620 nm with a microplate spectrophotometer. Dye content was normalized to tissue weight and expressed as micrograms of Evans Blue per gram of lung tissue.

### Lung wet/dry weight ratio

The left lung was carefully excised, and excess blood was gently removed using filter paper. The wet weight was measured immediately using an analytical balance. Lung tissues were dried at 60 °C for 72 h to determine their dry weight. The wet-to-dry (W/D) ratio was computed as the quotient of wet weight and dry weight, serving as an indicator of pulmonary edema severity.

### Immunohistochemistry staining

Paraffin-embedded lung tissues were sliced into 4 μm sections, deparaffinized in xylene, and rehydrated through a graded ethanol series (95%, 85%, 75%). To quench endogenous peroxidase activity, sections were treated with 3% hydrogen peroxide for 10 min at room temperature. Antigen retrieval was conducted by heating in 10 mM sodium citrate buffer (pH 6.0) for 15 min. Tissue sections were permeabilized with 0.1% Triton X-100 for 15 min and then blocked in 2% bovine serum albumin (BSA) for 30 min at room temperature. Subsequently, the slides were incubated overnight at 4 °C with primary antibodies against 4-HNE (ab46545, Abcam, 1:500). After washing with PBS, immunoreactivity was visualized using the REAL EnVision Detection System (HRP-labeled polymer, DAB+ chromogen; K5007, Dako/Agilent) according to the manufacturer’s instructions. Images were acquired using a bright-field microscope (Nikon, Japan).

### Immunofluorescence staining

For immunofluorescence staining, cells were fixed with 4% paraformaldehyde, permeabilized with 0.1% Triton X-100 for 15 min, and blocked in 2% BSA for 30 min at room temperature. Samples were incubated overnight at 4 °C with primary antibodies against F4/80 (ab6640, Abcam, 1:100), followed by incubation with Alexa Fluor 488-conjugated goat anti-rat IgG (H + L), cross-adsorbed secondary antibody (Invitrogen, A-11006; 1:1000) for 1 h at room temperature. Nuclei were counterstained with DAPI for 5 min. Images were acquired using a fluorescence microscope (Nikon, Japan).

### Bioinformatic analysis

Transcriptomic data of human AMs were obtained from the Gene Expression Omnibus (GEO) database (accession number GSE40885). Differential expression analysis between LPS-treated and control samples was performed using the GEO2R online tool, which applies the limma package for linear model fitting and the Benjamini-Hochberg method for multiple testing correction. Genes with |log2 fold change | ≥ 0.58 and adjusted *P*-value < 0.05 were considered differentially expressed genes (DEGs). The DEG results were subsequently imported into R (version 4.5.0) for visualization. A volcano plot illustrating the distribution of DEGs was generated using the ggplot2 package (Fig. 1A). This study did not involve the recruitment of human participants; only de-identified, publicly available transcriptomic data (GSE40885) were analyzed and therefore no additional institutional review board approval or informed consent was required. RNA-seq data from mouse AMs were generated following TRIzol-based RNA extraction, rRNA depletion, library preparation, and sequencing on an Illumina NovaSeq 6000 platform. Clean reads were aligned to the mouse reference genome (GRCm38) using HISAT2, and gene expression was quantified with StringTie; differentially expressed genes (DEGs) were defined as those with |log2FC | ≥ 0.58 and adjusted P-value < 0.05. GO and KEGG enrichment analyses were conducted in R 4.5.0 using the clusterProfiler package, and data visualization was performed with ggplot2; unless otherwise specified, an FDR < 0.05 was considered statistically significant.

### Statistical analysis

The sample size for the in vivo ARDS experiments was determined using an a priori power analysis conducted with G*Power software (version 3.1.9.7). The calculation was based on preliminary pilot data for the primary in vivo endpoints (lung injury score and lung wet/dry weight ratio) in the LPS-induced ARDS model, which indicated a large effect size (Cohen’s d = 1.8) and low inter-animal variability (coefficient of variation < 15%). To ensure adequate power to detect a significant effect in the primary in vivo ARDS experiment, we set the alpha (α) level to 0.05 (two-tailed) and statistical power (1−β) to 80%. Power calculations were performed for a two-group comparison (two-tailed unpaired t-test) based on the primary endpoint readouts. The analysis indicated that a minimum sample size of n = 4 mice per group would be sufficient. To account for potential biological variability and experimental loss, we used *n* = 4–6 mice per group for animal experiments (exact n values are provided in each figure legend). For in vitro assays, experiments were performed with at least three independent biological replicates, and technical replicates (if any) were averaged to generate a single value per biological replicate. No animals, samples, or data points were excluded from the analyses. Investigators performing viral administration, histological lung injury scoring, and quantitative analyses of immunoblots and immunofluorescence images were blinded to group allocation.

Statistical analyses were performed using SPSS version 23.0 and GraphPad Prism 8.0. Results are expressed as mean ± standard deviation (SD) unless otherwise indicated; non-parametric data (e.g., lung injury scores) are presented as median (interquartile range, IQR). Normality was assessed using the Shapiro–Wilk test, and homogeneity of variances was assessed using Levene’s test. For comparisons involving more than two groups, one-way ANOVA was applied, followed by appropriate post hoc testing (Tukey’s multiple-comparison test). Non-parametric data, such as lung injury scores, were evaluated using the Kruskal–Wallis test followed by Dunn’s multiple-comparison test. Unless otherwise indicated, all experiments were conducted independently at least three times. Statistical significance was defined as *p* < 0.05, based on a 95% confidence interval.

## Supplementary information

Supplementary Figures

Supplementary materials

## Data Availability

All data are available from the corresponding author with reasonable request.
