# Supplementary material for: Sympathetic signaling activation alleviated acute respiratory distress syndrome via the HDC/SLC7A11 axis in lipopolysaccharide-induced macrophages
Source: Cell Death Discov. 2026 Jun 15;12:343. doi: 10.1038/s41420-026-03173-0 (PMC13493966; doi:10.1038/s41420-026-03173-0)

Original image：Fig 1D

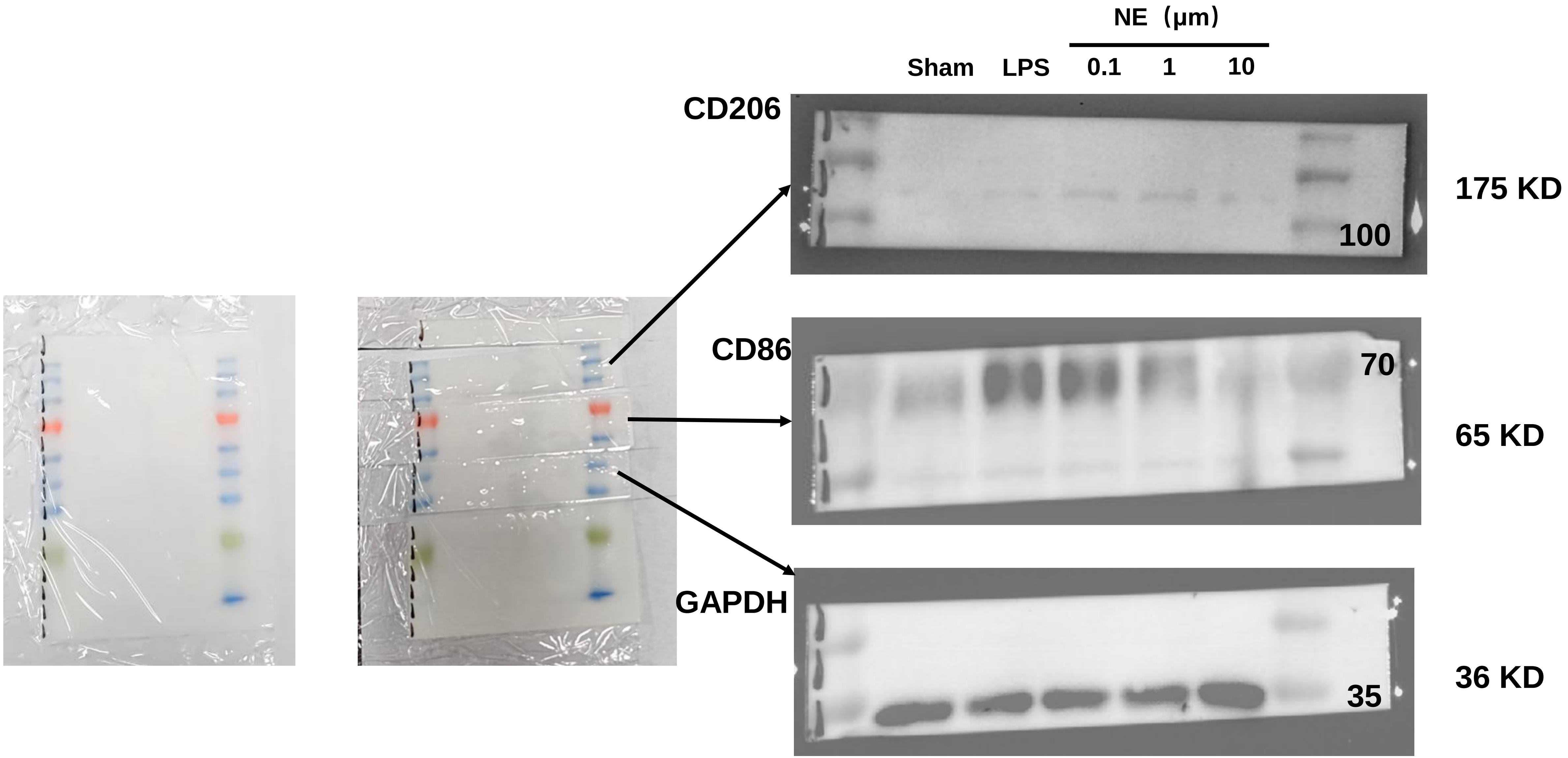

Original image：Fig 2D

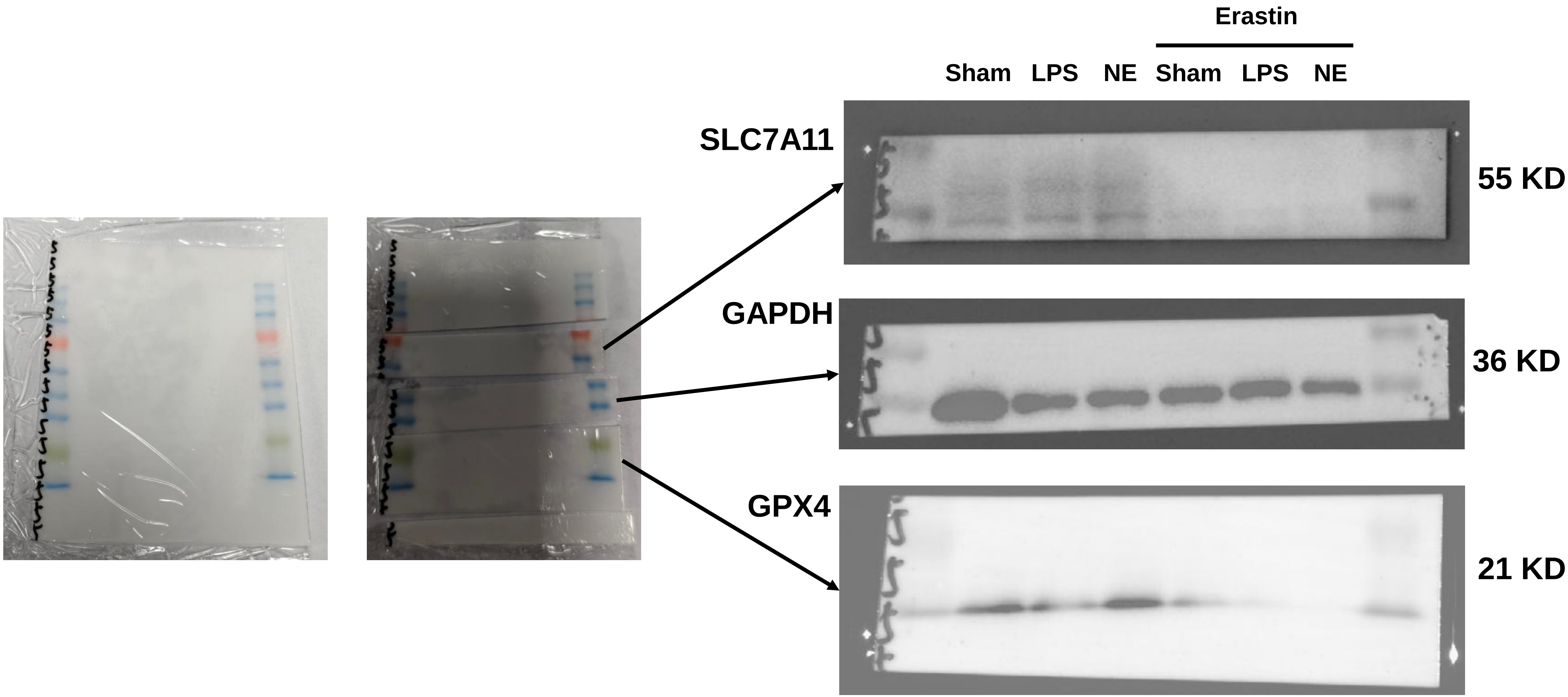

Original image：Fig 3D

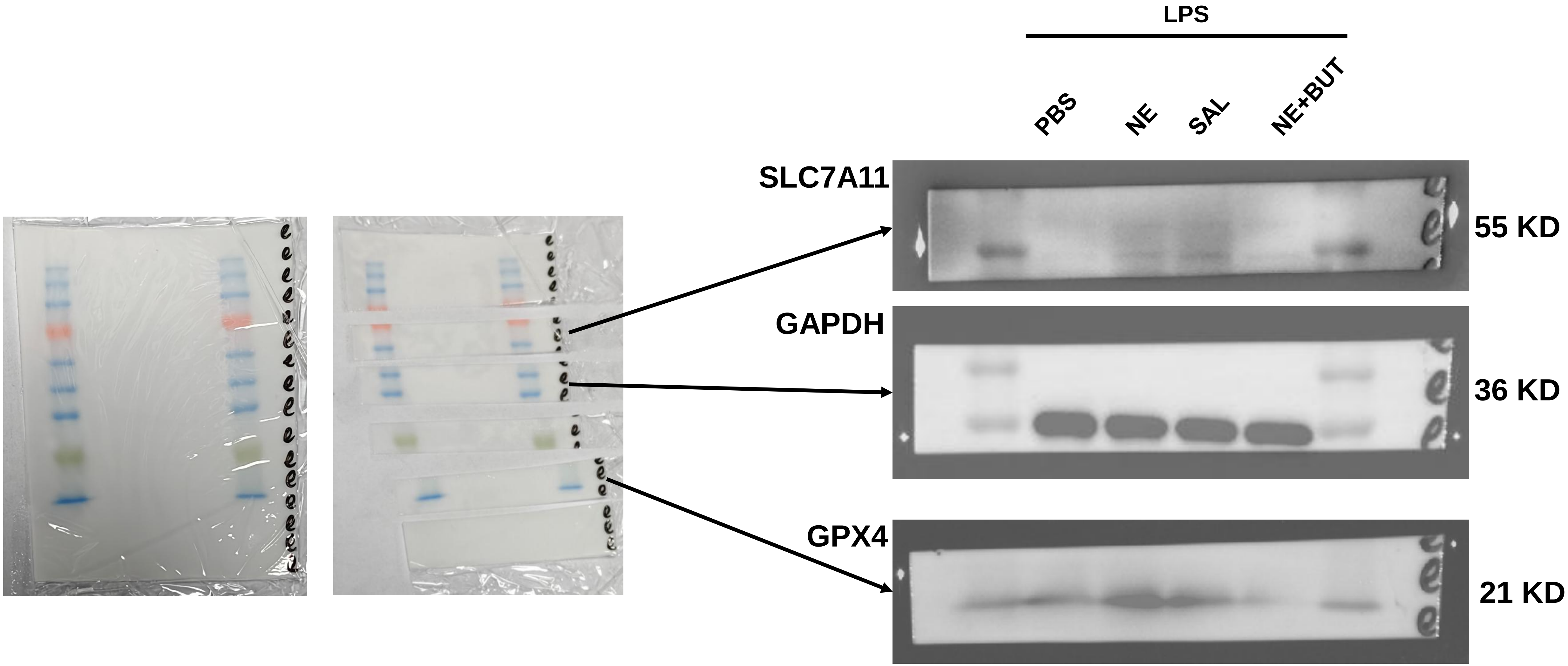

Original image：Fig 4F

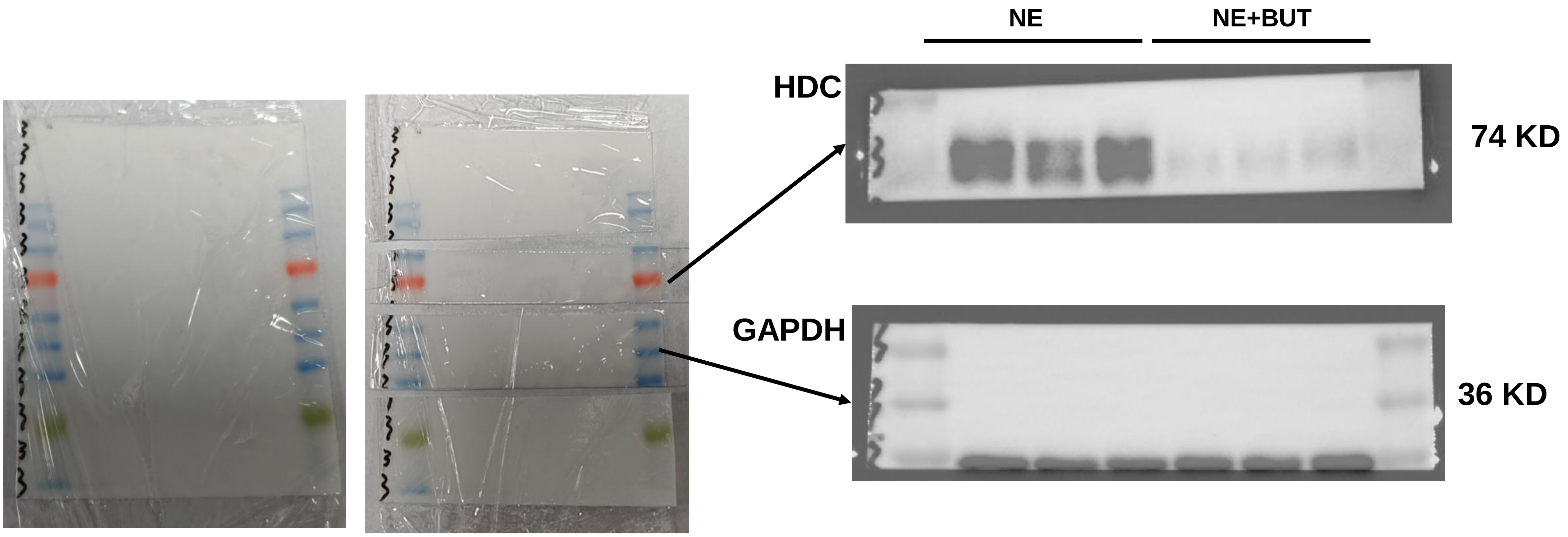

Original image：Fig 5I

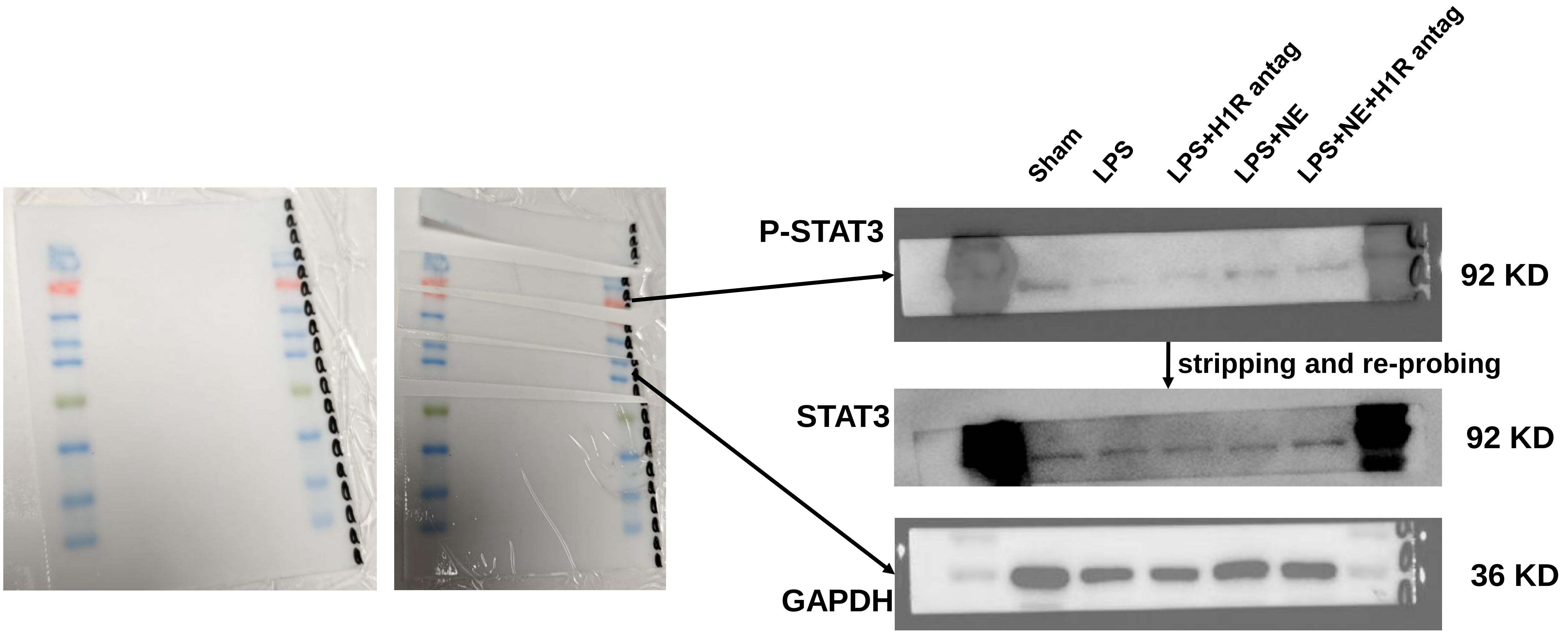

Original image：Fig 6H

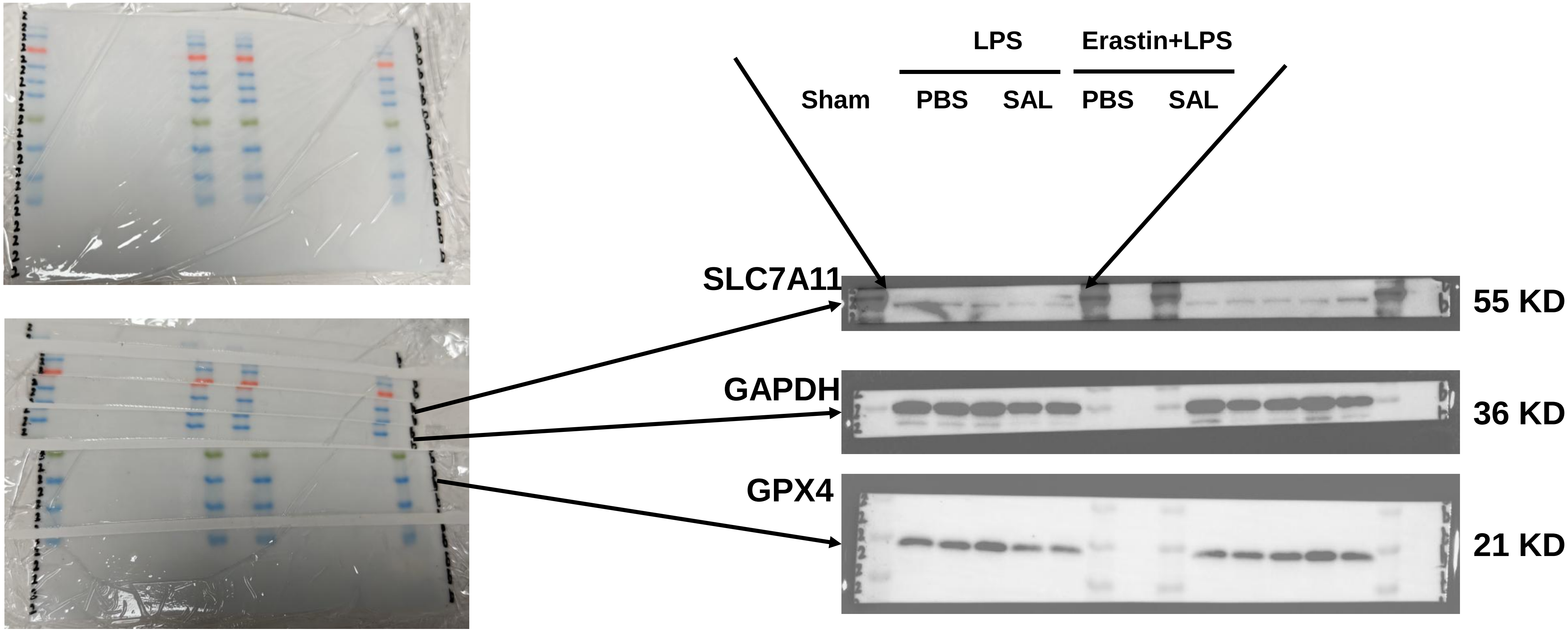

Original image：Fig S1A

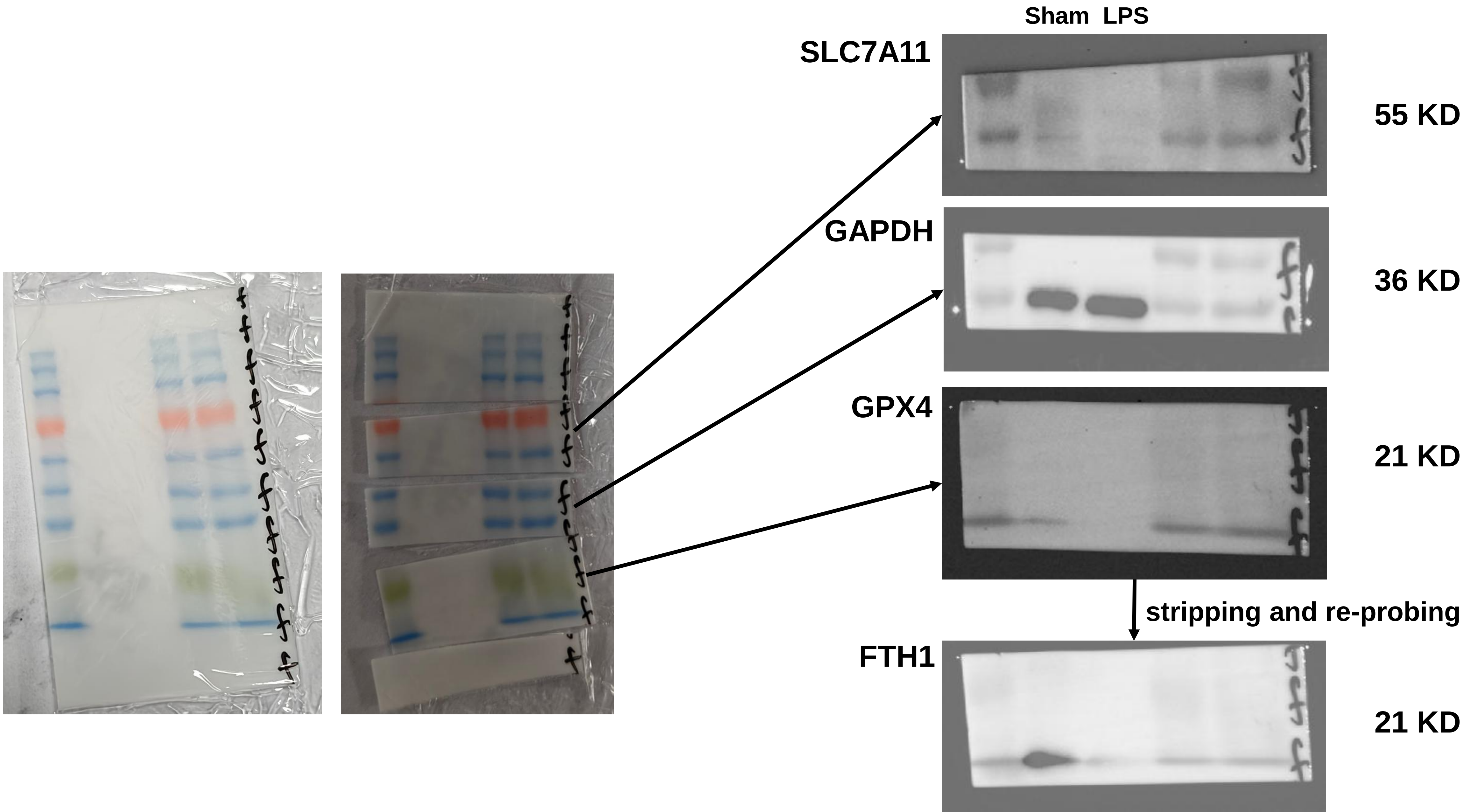

Original image：Fig S3C

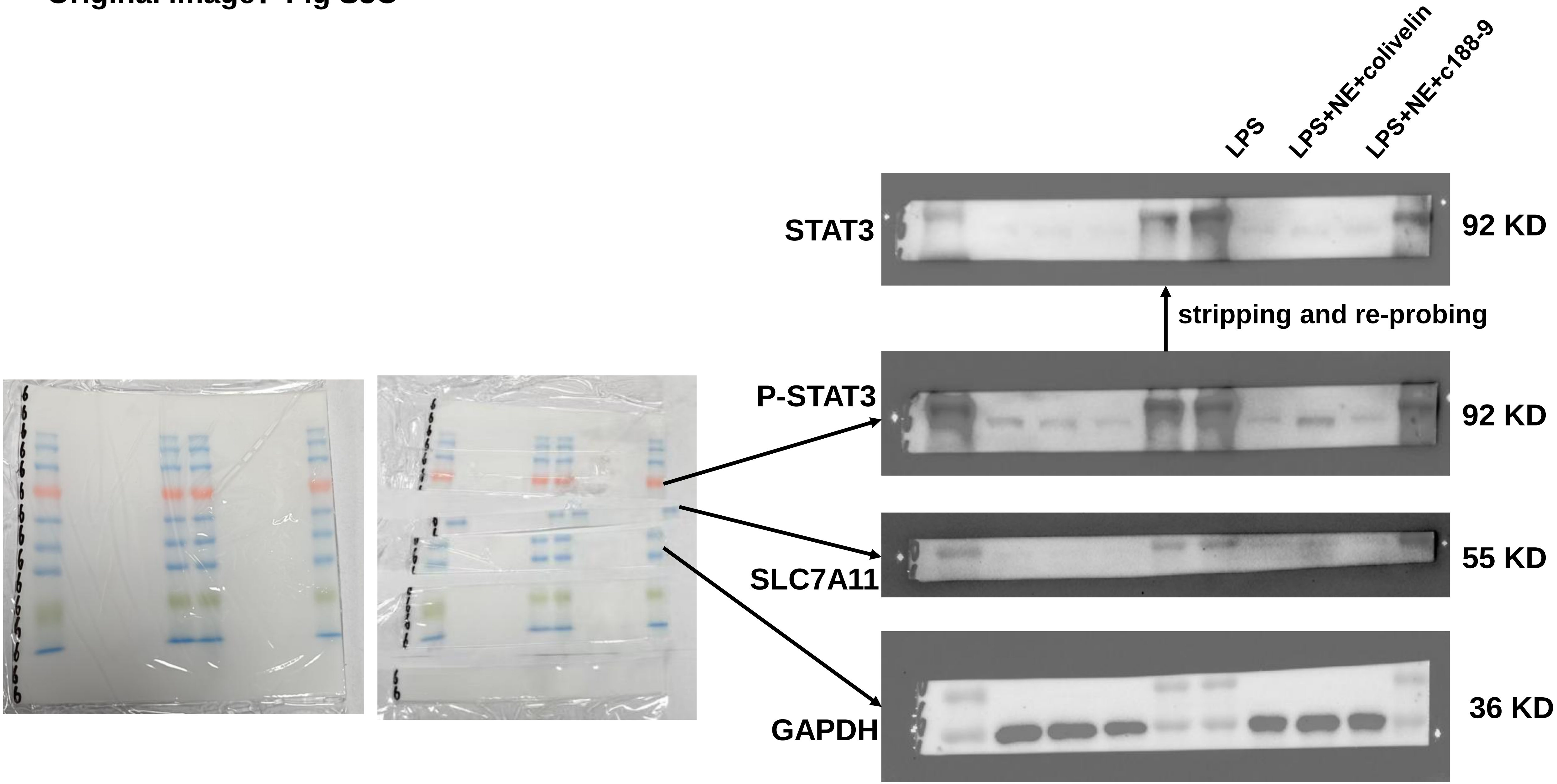

Original image：Fig S5C

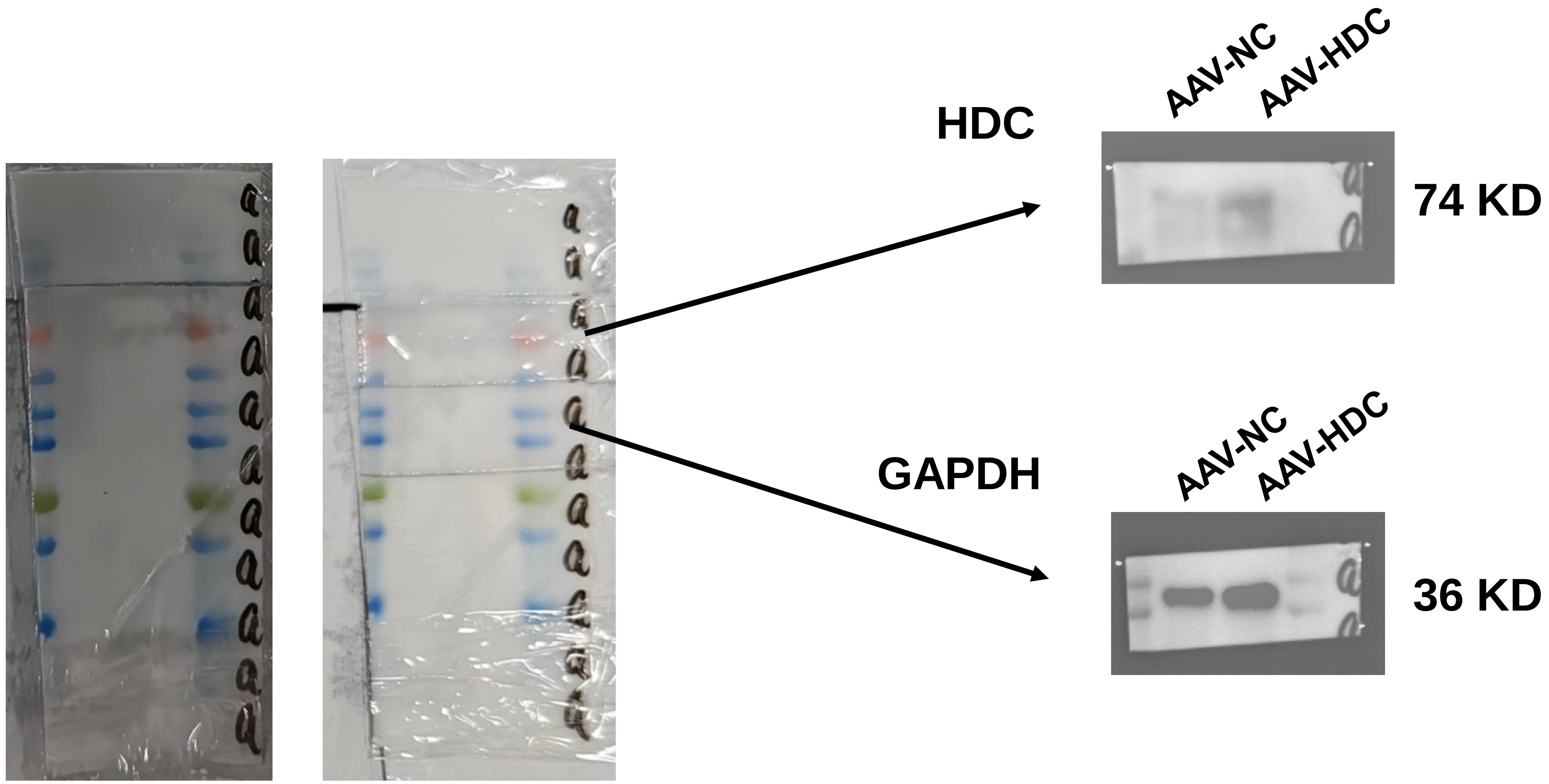

Supplement: Supplementary file 1 [file 41420_2026_3173_MOESM2_ESM.pdf]
