# Supplementary material for: Sympathetic signaling activation alleviated acute respiratory distress syndrome via the HDC/SLC7A11 axis in lipopolysaccharide-induced macrophages
Source: Cell Death Discov. 2026 Jun 15;12:343. doi: 10.1038/s41420-026-03173-0 (PMC13493966; doi:10.1038/s41420-026-03173-0)
Supplement: Supplementary file 2 [file 41420_2026_3173_MOESM1_ESM.docx]

This file contains supplementary **Figures S1-S6**, which include flow cytometry, immunofluorescence, Western blots, and ELISA data validating alveolar macrophage purity, demonstrating LPS-induced ferroptosis, and exploring the roles of β2-AR and STAT3 signaling and HDC overexpression in this process.

**Fig. S1 Validation of primary alveolar macrophage purity. (A)** Representative flow-cytometry plots of BALF cells from WT (left three samples) and intranasal LPS-induced ARDS mice (right three samples), showing Siglec-F⁺CD11c⁺ AMs accounting for >90% of viable BALF-derived cells in each sample. **(B)** F4/80 immunofluorescence of adherent BALF-derived cells from intranasal LPS-induced ARDS mice; nuclei were counterstained with DAPI. **(C)** Wright–Giemsa and Diff-Quik staining of BALF cytospin preparations from intranasal LPS-induced ARDS mice.

**Fig. S2 LPS triggers ferroptosis in AMs. (A–D)** Western blots for SLC7A11, FTH1, and GPX4 in AMs (n=4). **(E–G)** Relative mRNA levels of SLC7A11, FTH1, and GPX4 in AMs (n=10). Intracellular MDA**(H)** and Fe²⁺**(I)** contents in AMs (n=6). Data are presented as mean ± SD. **p<0.01, ***p<0.001, ****p<0.0001.

**Fig. S3 β2-adrenergic signaling mediates the anti-inflammatory effect of norepinephrine in AMs. (A)** ELISA analysis of TNF-α in AMs treated with norepinephrine (NE) and β2-AR antagonist butoxamine (BUT) (n=5). **(B)** ELISA analysis of IL-6 in AMs treated with norepinephrine (NE) and β2-AR agonist salbutamol (SAL) (n=5). Data are presented as mean ± SD. *p<0.05, **p<0.01, ***p<0.001, ****p<0.0001, ns, not significant.

**Fig. S4 Evidence of in vivo HDC overexpression in lung tissue and AMs** **(21 days after AAV administration). (A)** Representative immunofluorescence images of lung sections from mice receiving intratracheal AAV-HDC or control AAV (21 days post-injection). EGFP (green) indicates expression of the F4/80 promoter–driven AAV-HDC-P2A-EGFP transgene in the alveolar region; nuclei were counterstained with DAPI (blue). Scale bar: 1 mm (middle panels), 100 μm (bottom panels). **(B)** Relative mRNA expression of HDC in AMs from AAV-HDC and AAV-NC groups (n=5). **(C)** Western blots for HDC in AMs. Data are presented as mean ± SD. **p<0.01.

**Fig. S5 STAT3 signaling is required for norepinephrine-induced SLC7A11 expression and ferroptosis inhibition. (A)** MDA levels in LPS-treated AMs with norepinephrine plus colivelin or C188-9 (n=6). **(B)** Relative mRNA expression of SLC7A11 under indicated conditions (n=6). **(C)** Representative Western blots of P-STAT3, STAT3, SLC7A11, and GAPDH. **(D, E)** Quantification of SLC7A11 expression and P-STAT3/STAT3 ratio relative to GAPDH (n=5). Data are presented as mean ± SD. *p<0.05, **p<0.01, ***p<0.001, ****p<0.0001.

**Fig. S6 Densitometric analysis of GPX4 and SLC7A11 expression in lung tissue** **(related to Fig. 6H). (A, B)** Relative protein expression levels of GPX4 and SLC7A11 to GAPDH (n=5). Data are presented as mean ± SD. *p<0.05, **p<0.01, ***p<0.001, ****p<0.0001.
